# Identification of Inhibitors of the *Leishmania* cdc2-Related Protein Kinase CRK3

**DOI:** 10.1002/cmdc.201100344

**Published:** 2011-09-13

**Authors:** Laura A T Cleghorn, Andrew Woodland, Iain T Collie, Leah S Torrie, Neil Norcross, Torsten Luksch, Chido Mpamhanga, Roderick G Walker, Jeremy C Mottram, Ruth Brenk, Julie A Frearson, Ian H Gilbert, Paul G Wyatt

**Affiliations:** [a] Drug Discovery Unit, College of Life Sciences, James Black Centre, University of DundeeDundee, DD1 5EH (UK), Fax: (+44) 1382 386373 E-mail: p.g.wyatt@dundee.ac.uk; [b] Wellcome Centre for Molecular Parasitology and Division of Infection & Immunity, Faculty of Biomedical and Life Sciences, University of Glasgow120 University Place, Glasgow, G12 8TA (UK)

**Keywords:** CRK3, cyclin-dependent cdc2-related kinases, leishmaniasis, triazolopyridines, ureas

## Abstract

New drugs are urgently needed for the treatment of tropical parasitic diseases such as leishmaniasis and human African trypanosomiasis (HAT). This work involved a high-throughput screen of a focussed kinase set of ∼3400 compounds to identify potent and parasite-selective inhibitors of an enzymatic *Leishmania* CRK3–cyclin 6 complex. The aim of this study is to provide chemical validation that *Leishmania* CRK3–CYC6 is a drug target. Eight hit series were identified, of which four were followed up. The optimisation of these series using classical SAR studies afforded low-nanomolar CRK3 inhibitors with significant selectivity over the closely related human cyclin dependent kinase CDK2.

## Introduction

Neglected diseases are a major global cause of illness and death worldwide.[1] Kinetoplastid parasites, the causative agents of leishmaniasis, African sleeping sickness, and Chagas’ disease are a major cause of morbidity and mortality in developing countries. Approximately 350 million people are estimated to be at risk of leishmaniasis, with an estimated 12 million people infected worldwide.[2] Despite this disease burden, there is a lack of validated drug discovery targets and lead compounds for these neglected diseases.[1] Current therapeutic options are limited and unsatisfactory; therefore, there is an urgent need to discover new drugs for the treatment of major tropical parasitic diseases.

Kinases are well-known targets for a variety of diseases and disorders,[3] but they are underexplored as targets for neglected diseases. Kinetoplastid parasites have a significant kinome (approximately 190 protein kinases) with major differences from the human kinome (table S1, Supporting Information). The parasites have a large number of kinases involved in cell-cycle control, and this may be a reflection of the complex life cycle of these organisms. Cyclin-dependent cdc2-related kinases (CRKs) are the most investigated kinase family in this area.[4–7] The parasitic cyclin-dependent cdc2-related serine/threonine protein kinase (CRK3) has been postulated as a potential drug target for kinetoplastid species.[4] The CRK3–cyclin 6 complex (CRK3–CYC6) is thought to have homologous function to the cyclin-dependent kinase 1–cyclin B complex (CDK1–CYCB) in humans,[8, 9] which is essential for proliferation and coordination of the eukaryotic cell cycle. Gene disruption studies carried out in *Leishmania mexicana* to determine the necessity of CRK3 demonstrated that both CRK3 alleles can be replaced, although ploidy changes occurred to allow retention of the wild-type copy of CRK3, suggesting, but not confirming, that CRK3 is an essential enzyme for transition through the G_2_/M phase checkpoint of the *Leishmania* cell cycle responsible for parasite growth and survival.[8, 10]

Many human CDK inhibitors have been developed and are currently undergoing clinical trials despite the fact that the CDKs inhibited by these agents can be genetically knocked out without apparent major phenotypic changes.[9] This highlights the need for chemical as well as genetic validation. *Leishmania* CRK3–CYC6 inhibitors with micromolar potency were recently reported by Walker et al., following a high-throughput screen with heterocyclic and kinase libraries.[11] Grant et al. previously described the screening of a diverse chemical library of antimitotic compounds for potential inhibitors of *Leishmania* CRK3.[12] Although relatively successful, the broad-spectrum inhibitors identified failed to show selectivity over the mammalian CDK1–CYCB complex, and were in many cases equally or more potent against CDK1.[12]

The aim of this study was to identify novel and selective small-molecule inhibitors of *Leishmania* CRK3 to act as chemical probes for investigating the essentiality of *Leishmania* CRK3. There are multiple *Leishmania* CDKs and cyclins, and each kinase can form an active enzyme complex with more than one cyclin. This study focussed on the *L. mexicana* CRK3–*L. major* CYC6 complex (the *L. mexicana* CRK3 gene is 99 % similar to that of *L. major*), although other CRK–cyclin complexes could be potential targets. Compounds were counter-screened against the human cyclin-dependent kinase CDK2–cyclin A (CYCA) complex. This was used instead of CDK1–CYCB, the closest homologue of CRK3, as in our experience, CDK2 has proven to be a more promiscuous enzyme than CDK1, making it a more rigorous measure of selectivity. To check for general toxicity, a mammalian cell line was used as a secondary counter-screen.

Earlier we reported the assembly of a focussed set of potential kinase inhibitors to identify chemical starting points for kinase inhibitor discovery.[13] This focussed set of kinase inhibitors was screened against *Leishmania* CRK3–CYC6 to discover leads that can be optimised into suitable probes to chemically validate CRK3 as a drug target.

## Results and Discussion

### Binding site analysis of *Leishmania* CRK3

**Figure 1:**
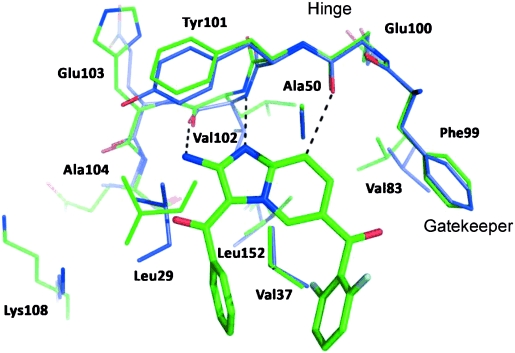
Superposition of the ligand binding sites *Leishmania* CRK3 (blue carbon atoms) homology model with a crystal structure of *Hs*CDK2 (green carbon atoms, PDB code 1PYE[19]) in complex with a ligand (bright-green carbon atoms). Residues Phe 82, Leu 83, His 84, and Gln 85 in CDK2 are respectively replaced with Tyr, Val, Glu, and Ala for *Leishmania* CRK3. The construction of the homology model and methods used for visualisation are described in the Experimental Section.

In the absence of crystal structures of the kinetoplastid CRK3s, a homology model of *L. mexicana* CRK3 was built by using human CDK2 (*Hs*CDK2) as the template. Analysis of the ATP binding sites revealed small differences between *Hs*CDK2 and *Leishmania* CRK3 ( Figure 1). The main divergence in amino acid side chains facing the ligand is the respective replacement of Phe 82 and Leu 83 in *Hs*CDK2 with tyrosine and valine. In addition, Gln 85 is replaced with alanine in *Leishmania* CRK3, and His 84 with glutamate. However, the latter changes are present for amino acids where the side chains are oriented away from the ligand binding site, and therefore these probably have only a minor effect on binding. This analysis shows that subtle changes could be explored to achieve selective inhibition of *Leishmania* CRK3 over *Hs*CDK2.

### Primary screen of focussed kinase library

**Figure 2:**
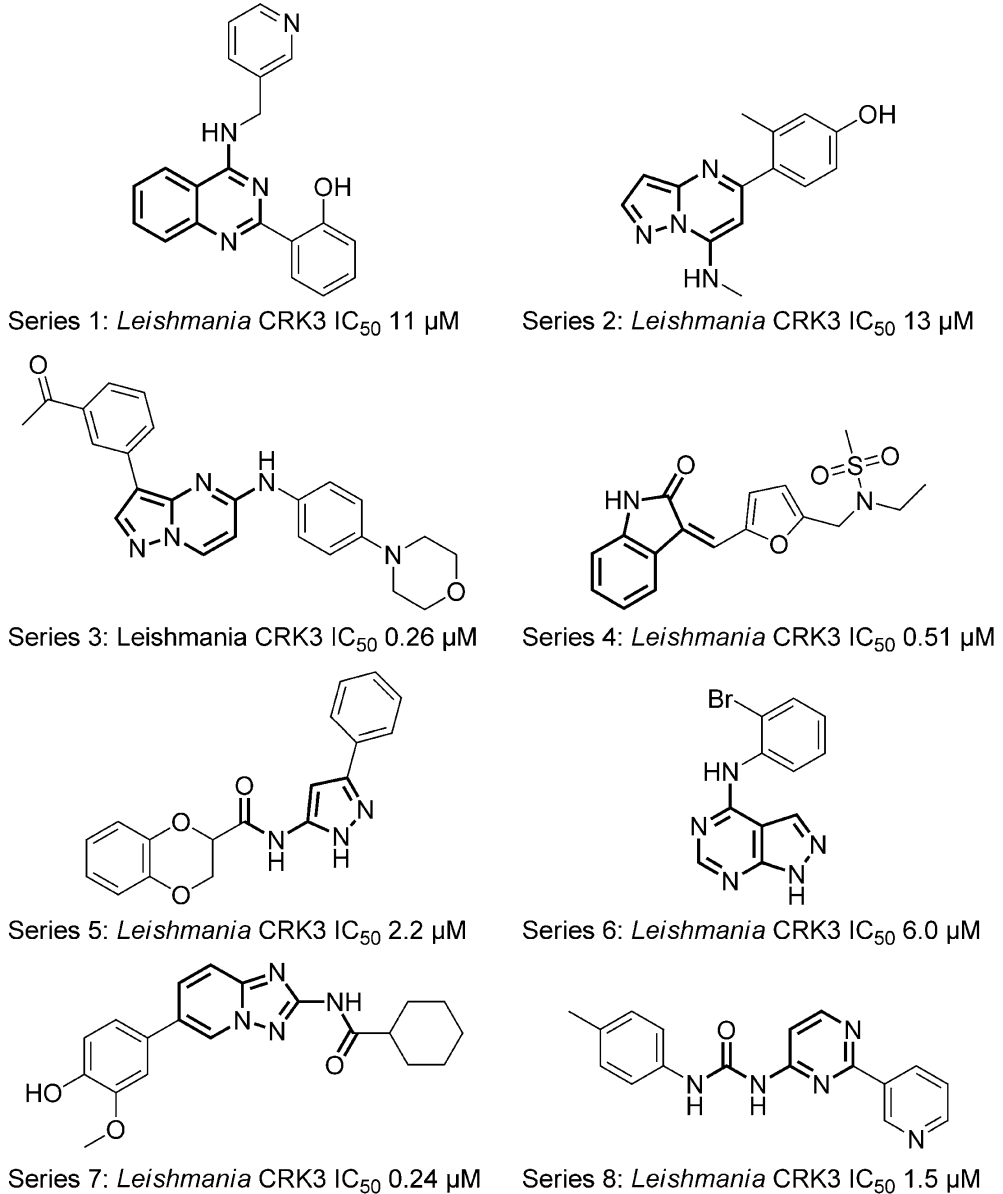
Representative compounds from hit series identified by screening the DDU focussed kinase set against the *Leishmania* CRK3–CYC6 complex using an IMAP assay platform with fluorescence polarisation detection, as described in the Experimental Section. The common substructure for each series is shown in bold.

Our in-house kinase library containing 3383[12] compounds was screened against *Leishmania* CRK3–CYC6 at a concentration of 30 μm. The 11 primary assay screen plates generated a robust mean (±SD) *Z′* value of 0.77 (±0.04) and a mean staurosporine potency (95 % confidence interval) of 29 nm (27–32 nm). The screen identified 73 compounds with inhibition values of ≥40 %, with 40 % representing a statistically significant threshold (>3×SD of the mean of the uninhibited control signal across all screening plates) for hit identification. These compounds were progressed into potency determination studies using 10-point dilution curves. Of these, 46 compounds gave IC_50_ values of ≤30 μm, with the most potent compound returning an IC_50_ value of 0.24 μm. Analysis of the whole data set identified eight compound series ( Figure 2) and seven singletons of interest (data not shown). Because the primary screen (IMAP assay—see *Enzyme assays* in the Experimental Section) conditions used >10 % of substrate during the course of the reaction, the potency of hits was reconfirmed with an orthodox “gold standard” radiometric secondary assay platform for *Leishmania* CRK3–CYC6. Initial selectivity was assessed using a similar radiometric *Hs*CDK2 assay. The secondary assays were run at an ATP concentration of <*K*_M_ for ATP, such that the IC_50_ value measured approximates to *K*_i_, thereby providing an accurate assessment of selectivity. Despite the 100-fold decrease in ATP substrate concentration between the IMAP-FP assay and the radiometric assay resulting in an apparent increase in potency values, there was a good correlation of potency values between the two platforms, and the rank order of hit potencies was retained (figure S1, Supporting Information). Mean staurosporine potency (95 % confidence interval) in the radiometric *Leishmania* CRK3 assay was 9.1 nm (8.0–10.5 nm) and 0.36 nm (0.31–0.43 nm) for *Hs*CDK2. The structure and purity of the hits taken into potency determinations were confirmed using LC–MS, and key compounds were re-synthesised to confirm the initial potency data. Hit series 3, 5, 7, and 8 were followed up in hit-to-lead studies, and the structure–activity relationship (SAR) results are reported herein. Series 1 and 2 were not pursued further due to lack of potency, series 6 was more potent against the *Hs*CDK2 counter-screen (IC_50_=1 μm) than *Leishmania* CRK3, and series 4 was not pursued further due to the potential Michael acceptor moiety.

#### Compound series 3

The primary screen identified a range of pyrazolo[1,5-*a*]pyrimidines (series 3, Figure 2) with modifications at both the 3- and 7-positions. The 35 compounds tested with aromatic substituents at the 3-position and diverse structural types from the amine at the 7-position gave inhibition values ranging from 0 to 93 % at 30 μm, suggesting that SAR could be derived in this series. To confirm the data obtained from the screening samples, a further array of compounds was synthesised (table S2, Supporting Information); these, however, displayed only moderate potency and poor selectivity over *Hs*CDK2. When an example from the series was profiled against a panel of 76 human kinases, it proved to be a nonselective inhibitor with >50 % inhibition at 10 μm for 47 of these human kinases. Due to the lack of selectivity for parasite kinases over host kinases and activity not being translated into cells in culture, this series was not developed further (table S4, Supporting Information).

#### Compound series 5

**Figure 3:**
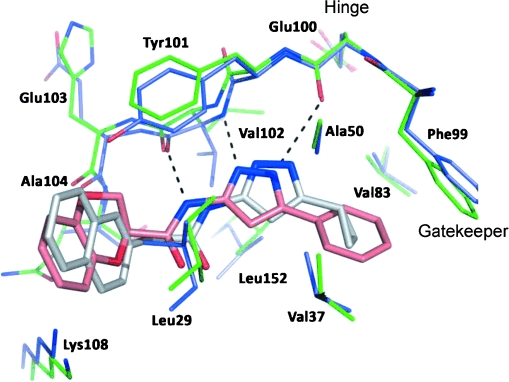
Crystal structure of a pyrazole-based inhibitor (grey carbon atoms) of *Hs*CDK2 (PDB code 1VYW, green carbon atoms)[12] superimposed with the modelled binding mode of **6** (pink carbon atoms) bound to *Leishmania* CRK3 (blue carbon atoms).

The set of focussed kinase inhibitors contained only seven examples of the aminopyrazole template (series 5, Figure 2), and these showed interesting levels of activity and potential SAR patterns ( Figure 2 and Table 1, compounds **1**–**6**). A crystal structure of *Hs*CDK2 in complex with a pyrazole amide scaffold-based inhibitor was published previously (PDB code 1VYW).[12] Based on this structure, binding modes for pyrazole amides bound to *Leishmania* CRK3 were modelled ( Figure 3). It was assumed that the pyrazole amide scaffold adopts the same binding mode in *Leishmania* CRK3 as in *Hs*CDK2 with all nitrogen atoms forming hydrogen bonds with the amino acid backbone of the hinge region. As a consequence, the substituents at the R^1^ position are oriented towards the gatekeeper residue (Phe 80), and substituents at the R^2^ position within a lipophilic pocket are directed out towards the solvent (R^1^ and R^2^ group definitions are listed in Table 1).

**Table 1:** *Leishmania* CRK3–CYC6 and *Hs*CDK2–CYCA activity of pyrazoles **1–11** (Series 5)

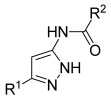					
Compd	R^1^	R^2^	CRK3–CYC6 Inhib. [%][a]	CRK3–CYC6 IC_50_ [μm][b]	*Hs*CDK2–CYCA IC_50_ [μm][c]
**1**	3,5-dimethoxyphenyl	methyl	0	–	–
**2**	4-chlorophenyl	methyl	74	3.0	1.6
**3**	phenyl	methyl	17	–	–
**4**	phenyl	4-methoxyphenyl	55	2.4	–
**5**	phenyl	2-(2,3-dihydrobenzo[*b*][1,4]dioxin	86	0.65	1.6
**6**	methyl	4-phenoxyphenyl	7	–	–
**7**	4-chlorophenyl	cyclobutyl	99	0.9	1.2
**8**	4-chlorophenyl	benzyl	88	3.0	0.49
**9**	4-chlorophenyl	phenyl	44	>100	>100
**10**	4-chlorophenyl	3-pyridyl	100	2.4	5.1
**11**	4-chlorophenyl	cyclohexyl	100	0.34	>100

[a] Percent inhibition of *Leishmania* CRK3–CYC6 activity at 30 μm.

[b] Concentration required to inhibit *Leishmania* CRK3–CYC6 activity by 50 %; data represent the mean of two or more experiments.

[c] Concentration required to inhibit CDK2–CYCA activity by 50 %; data represent the mean of two or more experiments.

The model appears to rationalise the binding data. Thus a 3,5-dimethoxyphenyl substituent of the pyrazole **1** (Table 1) showed poor activity, presumably due to the inability of the gatekeeper residue position to accommodate a group of this size. The more linear 4-chlorophenyl group of compound **2** can be accommodated with an increased affinity relative to **3**, probably due to improved edge–face interactions of the electron-poor system with the gatekeeper. On the other side of the active site, the lipophilic pocket can bind the phenyl amide of **4** and the bicyclic moiety of **5**, whereas larger linear groups such as the phenoxybenzamide of **6** led to a complete loss of binding affinity. This observation is consistent with the binding mode of pyrazole amides in *Hs*CDK2 and previously established SAR data ( Figure 3),[14, 15] where the most potent *Hs*CDK2 inhibitors in this series contain an sp^3^ carbon atom next to the amide group which induces an sp^3^ bend in the molecule. This bend allows the accommodation of bulky substituents in this area of the binding site, whereas more linear moieties lead to a steric clash. Although *Leishmania* CRK3 and *Hs*CDK2 share high sequence identity, inhibitor selectivity between closely related human CDK family members is well known.[16–18] We therefore decided to pursue the preparation of compounds selective for the parasite kinase. A small set of compounds (**7**–**11**, Table 1) were synthesised to examine whether selectivity could be achieved. We focussed on modifying the 5-position substituent (R^1^) to investigate the interaction with the gatekeeper residue (Phe 80 in *Hs*CDK2), and the amide (R^2^) to probe the lipophilic pocket along the backbone of the kinase.

### Chemistry

**Scheme 1:**
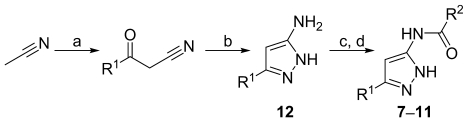
Synthesis of compounds **7**–**11**. *Reagents and conditions:* a) LiN(SiMe_3_)_2_, THF, −78 °C, 1 h, then addition of R_1_CO_2_Me; b) N_2_H_4_⋅H_2_O, EtOH, 70 °C, 24 h; c) R_2_COCl (1.1 equiv), 1,4-dioxane, 150 °C, 5 min (MW), then addition of PS isocyanate (2 equiv), 160 °C, 5 min (MW); d) 1:1 EtOAc/NaOH (2 m solution in H_2_O), RT, 1 h.

The synthesis of the heterocyclic core (Scheme 1) was carried out through condensation of acetonitrile with an appropriate ester to give 2-cyanoketones **12** in 26–94 % yield. Reaction of **12** with hydrazine hydrate in ethanol at reflux gave 5-substituted pyrazole-3-amines **13** in yields of 30–77 %. A general acylation method was developed that involved microwave heating and solid-supported scavengers; this allowed the rapid synthesis of 5-substituted 3-aminopyrazole-3-yl amides (Table 1, **7**–**11**). Amines **13** were treated with acid chlorides, and then excess **13** was scavenged using polymer-supported (PS) isocyanate resin. The crude products were treated with base to hydrolyze any over-acylated products. Extraction and concentration yielded the desired final compounds **7**–**11** in 2–80 % yield without the need for further purification in most cases.

Modification of the amide substituent was examined by using 4-chlorophenyl (R^1^) derivatives (Table 1, synthesised compounds **7**–**11**). Introduction of small carbocyclic rings (compound **7**) resulted in a threefold improvement in *Leishmania* CRK3 activity relative to the initial screening hit **2**, whereas extension to the corresponding benzyl derivative **8** had no effect. Increasing ring size to phenyl **9** from cyclobutyl **7** resulted in a complete loss of activity against both enzymes, a modification well tolerated for *Hs*CDK2 with the smaller cyclopropyl substituent at the 5-position.[12, 13] Interestingly, the 3-pyridyl amide **10** did not suffer from loss of activity, possibly through significant interaction either directly or through water-mediated hydrogen bonds. The cyclohexyl amide **11** afforded a 10-fold increase in activity against *Leishmania* CRK3, but more importantly, diminished *Hs*CDK2 activity, resulting in a compound with significant selectivity for *Leishmania* CRK3–CYC6, although we cannot explain the detailed reasons for this from our model.

As compound **11** was the most potent compound against *Leishmania* CRK3–CYC6 and highly selective over *Hs*CDK2, we decided to retain the cyclohexyl group on the amide and then optimise the substituent at position R^1^. Further modification of R^1^ of **11** either as cycloalkyl, substituted phenyl, heteroaryl, or benzyl did not afford an increase in *Leishmania* CRK3 activity (table S3, Supporting Information). Comparing the SAR generated within our studies with that reported for *Hs*CDK2 by Pevarello et al.[14, 15] suggested some subtle differences in the structure of *Hs*CDK2 and *Leishmania* CRK3, despite their close homology. Compound **11** proved to be highly selective against a panel of 76 human kinases, with only three kinases—ERK8, GSK3β and IRR—showing >50 % inhibition at 10 μm (table S5, Supporting Information).

#### Compound series 7

[1,2,4]Triazolo[1,5-a]pyridine **14** ( Figure 3) was identified as an initial hit from primary screening. Compound **14** had an IC_50_ value of 0.24 μm against *Leishmania* CRK3 but also inhibited with similar potency in the *Hs*CDK2 counter-screen (IC_50_=0.36 μm). [1,2,4]Triazolo[1,5-a]pyridine-2-ylamines are a novel structural scaffold for *Leishmania* CRK3 inhibition and none have been reported thus far for antiparasitic activity.

### Putative binding mode analysis

**Figure 4:**
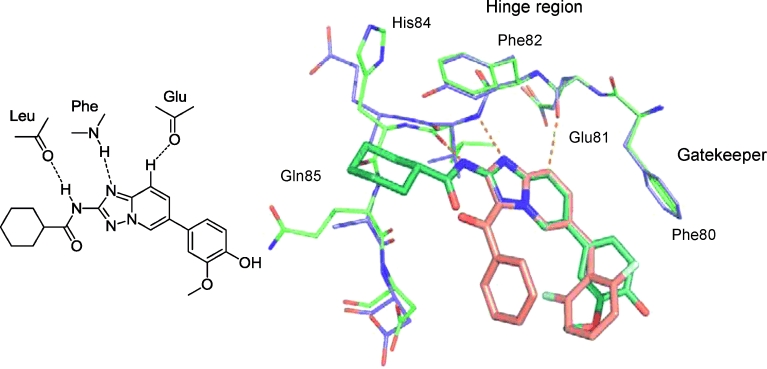
Overlap of the 5,6 bicyclic core of the known aminoimidazo[1,2-*a*]pyridine *Hs*CDK2 inhibitor (pink) with **14** (cyan). Dashed lines represent the proposed hydrogen bond interaction with the hinge region.

We carried out modelling studies to guide the synthetic programme. Triazolopyridine **14** was superimposed onto the known *Hs*CDK2 aminoimidazo[1,2-*a*]pyridine inhibitor ( Figure 4).[19] This was followed by energy minimisation in the binding site using the MAB force field (see the Experimental Section). The result was a binding mode that shows a classic kinase inhibitor hydrogen bond donor/acceptor binding pattern to the hinge region.[20] Although good alignment was observed for the core motifs, the substituents occupy different areas of the binding site. The proposed binding mode includes the following key interactions: a hydrogen bond between the amide N–H of **14** and the carbonyl group of Val 102 (*Hs*CDK2) (or Leu in *Leishmania* CRK3) ( Figure 4), the triazolopyridine nitrogen hydrogen bonded to the α chain N–H group of Phe 82 (*Hs*CDK2), and a possible interaction between the 6-phenyl aromatic hydrogen atom of **14** and the backbone carbonyl group of Glu 81 in both *Hs*CDK2 and *Leishmania* CRK3 (a C–H hydrogen bond). This putative binding mode was used as a starting point for the rational design of inhibitors exploring two main areas in the binding site: the hydrophobic gatekeeper region and the lipophilic pocket leading to solvent (the region next to Gln 85 in Figure 4). The substituents of the amide and the aromatic ring at the 6-position of the triazolopyridine scaffold **14** were investigated to optimise potency against *Leishmania* CRK3 and *Leishmania* in culture while introducing selectivity over *Hs*CDK2.

### Chemistry

**Scheme 2:**
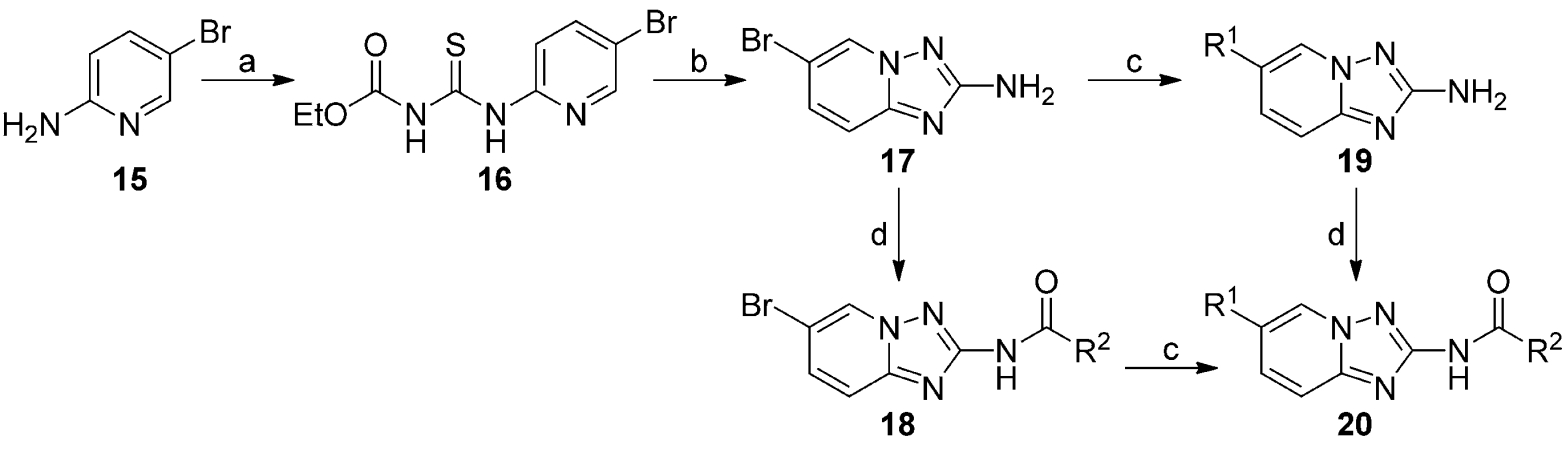
*Reagents and conditions:* a) Ethoxycarbonylisothiocyanate (1 equiv), dioxane (3 mL mmol^−1^), under argon, 0 °C→RT, 17 h; b) NH_2_OH⋅HCl (5 equiv), DIPEA (3 equiv), EtOH/MeOH 1:1 (3.76 mL mmol^−1^), 2 h RT then 3 h 60 °C; c) acid chloride (1 equiv), DMAP (0.1 equiv), pyridine (4 mL mmol^−1^), 0 °C→RT, 17 h;[27] d) boronic acid (1.2 equiv), DMF/2 m K_2_CO_3_ (1:1), Pd(PPh_3_)_4_ (0.01 equiv), 140 °C (MW), 15 min (see Experimental Section for specific reagents).

The initial synthetic strategy required the synthesis of the common intermediate 6-bromo-[1,2,4]triazolo[1,5-a]pyridine-2-amine (**17**). From this point, either R^1^ or R^2^ can be kept constant, enabling arrays to be performed by varying the other substitution vector (Scheme 2). Treatment of commercially available 2-amino-5-bromopyridine **15** with ethoxycarbonylisothiocyanate afforded the corresponding aminothiomethylethyl ester **16**. Cyclisation to give the triazolopyridine scaffold **17** was carried out by reaction with hydroxylamine hydrochloride in the presence of base.[19] Diversity can be introduced from this point; reaction of amine **17** with an acid or acid chloride affords amide **18**, which undergoes facile microwave-irradiated Suzuki reaction with a series of aryl boronic acids to give **20**. Reversal of the reaction order allows the R^1^ group to be kept constant, with variation of R^2^ (**17** to **19** to **20**).

### Enzyme activity

Initial work focussed on varying R^1^ (which we believe may interact with the gatekeeper residue) whilst maintaining R^2^ as cyclohexyl (Table 2). Removal of the aromatic ring was not tolerated, with loss of activity in both *Leishmania* CRK3 and *Hs*CDK2 with R^1^=H. A phenyl group at R^1^ (compound **22**) demonstrated that it is possible to achieve selectivity and retain potency. By examining the substituents on the initial hit **14** with 4-hydroxyphenyl **23** and 3-methoxyphenyl **24** separately, 4-hydroxy was found to be responsible for retention of *Hs*CDK2 activity, while monosubstituted 3-methoxy was inactive against *Hs*CDK2. Although not apparent in our analysis of the 3D models, this result implies that there is the potential for (water-mediated) hydrogen bonding to the hydroxy group, and that this is not essential for *Leishmania* CRK3 activity. 3-Methyl compound **25** was found to give the best potency and selectivity (0.064 μm *Leishmania* CRK3, >100 μm *Hs*CDK2) among those compounds investigated in the initial plan.

**Table 2:** *Leishmania* CRK3–CYC6 and *Hs*CDK2–CYCA activity (compounds 14–39) from initial SAR development at the R^1^ position of triazolopyridine 19

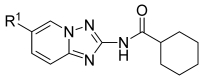			
Compd	R^1^	CRK3–CYC6 IC_50_ [μm][a]	*Hs*CDK2–CYCA IC_50_ [μm][b]
**14**	4-hydroxy-3-methoxyphenyl	0.23	0.36
**21**	H	>100	>100
**22**	phenyl	0.27	>100
**23**	4-hydroxyphenyl	0.12	0.86
**24**	3-methoxyphenyl	0.36	>100
**25**	3-methylphenyl	0.06	>100
**26**	3-chlorophenyl	0.35	>100
**27**	1-napthyl	0.09	>100
**28**	4-indole	0.07	>100
**29**	4-*N*-methylindole	0.04	>100
**30**	3-morpholinophenyl	1.6	>100
**31**	2,6-dimethylphenyl	19.0	>100
**32**	2,5-dimethylphenyl	0.65	>100
**33**	3,5-dimethylphenyl	0.02	>100
**34**	4-methoxy-3-methylphenyl	0.03	>100

[a] Concentration required to inhibit *Leishmania* CRK3–CYC6 activity by 50 %; data represent the mean of two or more experiments.

[b] Concentration required to inhibit *Hs*CDK2–CYCA activity by 50 %; data represent the mean of two or more experiments.

The homology model suggested that bulkier aromatic and hydrophobic R^1^ groups could be accommodated in the region of the pocket proximal to the gatekeeper. We postulated that potency could be increased by exploiting this space; therefore, further studies focussed on this hypothesis and developing the SAR around the 3-methylphenyl substituent (Table 2). 1-Napthyl **27**, 4-indole **28**, and 4-*N*-methyl indole **29** gave results similar to that of the 3-methyl, suggesting that there is a limited benefit. 3-Morpholine **30** was synthesised to explore the region above the gatekeeper for favourable and potentially selective polar interactions; complete selectivity for *Leishmania* CRK3 over *Hs*CDK2 was observed, but with only moderate enzyme activity. Finally an additive approach with disubstituted aromatics was examined: 2,6- and 2,5-dimethyl groups (**31** and **32**) were detrimental to activity; however, both 3,5-dimethyl **33** and 4-methoxy-3-methyl **34** showed a two- to threefold improvement in potency. These results imply a bulky aromatic group favours inhibition of *Leishmania* CRK3, and not *Hs*CDK2.

SAR of the amide moiety was examined by employing the previously discovered 3-methylphenyl or 3,5-dimethylphenyl as the R^1^ group (Table 3). The free amine **35** was inactive against *Leishmania* CRK3 but retained weak *Hs*CDK2 activity; removal of the amide carbonyl **36** retained only moderate *Leishmania* CRK3 activity and gave weak *Hs*CDK2 activity. Replacement of the lipophilic cyclohexyl group with a methyl substituent **37** showed a large drop in activity and similar levels of *Hs*CDK2 activity. 2-Tetrahydrofuran, 4-tetrahydropyran, and 3-piperidine (compounds **38**–**40**) were incorporated to explore the possibility of water-mediated interactions to residues such as Tyr, which replaces Phe 82 in the hinge region of *Leishmania* CRK3 ( Figure 4). Although these compounds retained selectivity, they showed low-micromolar *Leishmania* CRK3 activity. This set of compounds confirm that the lipophilic nature of the cyclohexyl substituent is important; replacement of the heteroatom with a carbon atom gives a minimal 10-fold increase in activity (compare compounds **38** and **39** with **43**, and **40** with **25**). These data imply the cyclohexyl substituent is in the lipophilic pocket as suggested by the model, giving an enhancement in potency commonly observed for *Hs*CDK2 inhibitors.[21] Rigidification of the cyclohexyl ring by replacement with a planar phenyl substituent retains selectivity, but results in a 10-fold loss in activity (compound **41**). Generally, increasing the ring size of the lipophilic probe increases the potency (Table 3, **42**–**48**). This affinity gain may be credited to a gain in entropy due to the displacement of water molecules from this region of the binding site. Insertion of a carbon linker between the amide and the cyclic alkyl group improved activity (**44**–**46**); methyl gave ∼2-fold and ethyl ∼30-fold improvements. This trend was also observed with the phenyl substituents [phenyl **41** (IC_50_=1.1 μm), phenethyl **47** (IC_50_=0.03 μm)]. Combining the optimal groups from the SAR studies of the aromatic and amide substituents gave the expected low-nanomolar activities (Table 3, **48**), showing that potency against *Leishmania* CRK3 can be improved to the low-nanomolar range whilst retaining selectivity over *Hs*CDK2.

**Table 3:** *Leishmania* CRK3–CYC6 and *Hs*CDK2–CYCA activity from initial SAR investigations at the R position of triazolopyridine 19 (Scheme 3) and a benzimidazole core

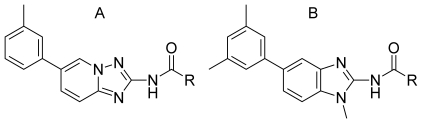				
Compd	Core	R	CRK3–CYC6 IC_50_ [μm][a]	*Hs*CDK2–CYCA IC_50_ [μm][b]
**35**	A	free NH_2_, no amide	>100	37
**36**	A	cyclohexylmethyl, no amide	3.0	64
**37**	A	methyl	65	26.0
**38**	A	2-tetrahydrofuran	12	>100
**39**	A	4-tetrahydropyran	10	>100
**40**	A	3-piperidine	12	>100
**41**	A	phenyl	1.1	>100
**42**	A	cyclobutyl	3.3	>100
**43**	A	cyclopentyl	1.1	>100
**44**	A	cyclopentylmethyl	0.04	>100
**45**	A	cyclohexylmethyl	0.01	>100
**46**	A	cyclopentylethyl	<0.005	>100
**47**	A	phenethyl	0.03	>100
**48**	B	cycloheptyl	<0.005	>100

[a] Concentration required to inhibit *Leishmania* CRK3–CYC6 activity by 50 %; data represent the mean of two or more experiments.

[b] Concentration required to inhibit *Hs*CDK2–CYCA activity by 50 %; data represent the mean of two or more experiments.

#### Kinase selectivity of compound series 7

Compounds **25**, **29**, **33**, and **34** were screened against the DSTT Dundee kinase panel (76 human kinases)[22] and were found to be highly selective. *Hs*CDK2–CYCA is in the Dundee human kinase panel, and none of the compounds showed inhibition of this, confirming the selectivity observed in our assays over the human orthologue. For **25**, **29**, **33**, and **34** the main kinases showing >50 % inhibition at 10 μm were DYR 1A, 2, 3, and IRR. Compound **25** showed a very clean profile, only mildly inhibiting MKK and IRR (table S6, Supporting Information). These results confirm that the compound series is highly selective for parasitic over human kinases, and does not afford broad-spectrum kinase inhibition. Compound **25** was tested against *L. major* wild-type promastigote cells, but limited activity was observed (IC_50_=2–10 μm).

#### Compound series 8

**Figure 5:**
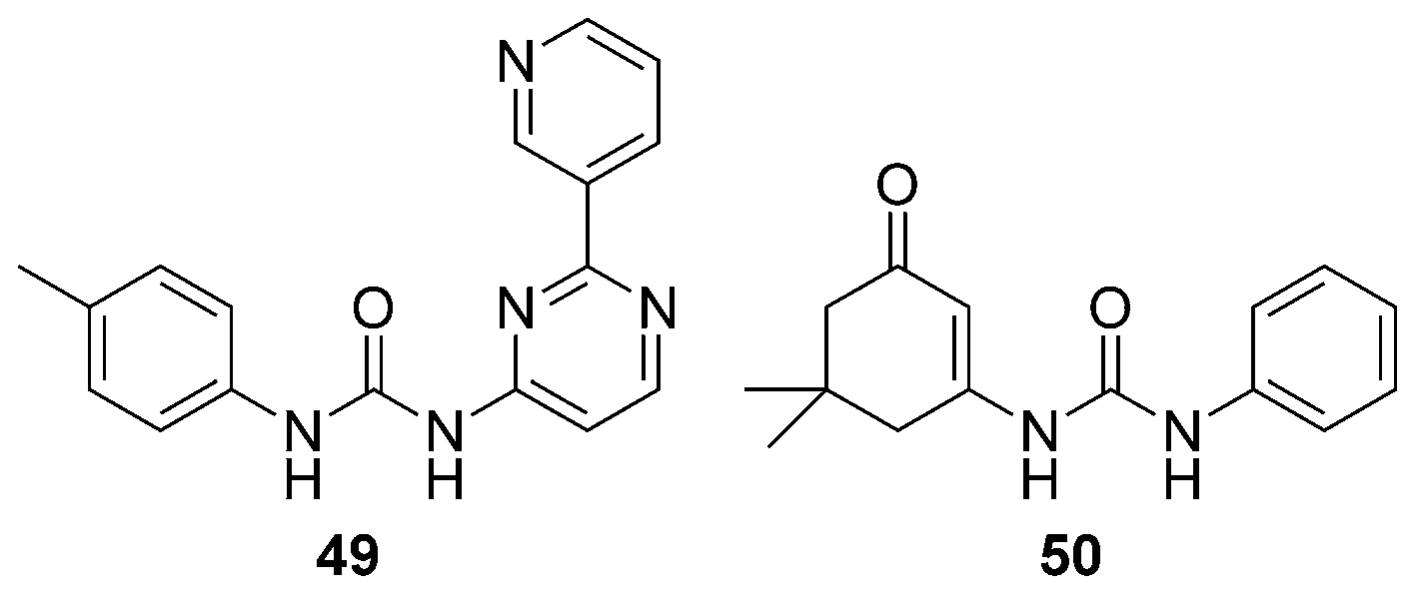
Series 8 urea leads. Compound **49**: *Leishmania* CRK3 IC_50_=1.5 μm, *Hs*CDK2–CYCA IC_50_=0.026 μm, MRC5 EC_50_=12 μm; compound **50**: *Leishmania* CRK3 IC_50_=0.16 μm, *Hs*CDK2–CYCA IC_50_=100 μm, MRC5 EC_50_≥50 μm.

Series 8 consisted of two structurally diverse disubstituted ureas ( Figure 5). Compound **49** displayed moderate *Leishmania* CRK3 enzyme activity, and did not inhibit *L. major* wild-type promastigote cells (EC_50_>50 μm); however, none of the modifications investigated increased the activity against *Leishmania* CRK3 or afforded any significant improvements in selectivity over *Hs*CDK2.

**Scheme 3:**
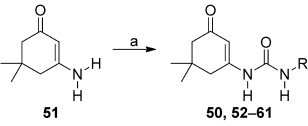
Synthesis of ureas **50**, **52**–**61**. *Reagents and conditions:* a) R–N=C=O, 1,4-dioxane, 90 °C, 12 h.

Compound **50**, with potent inhibition of *Leishmania* CRK3, excellent selectivity over *Hs*CDK2, and good predicted ADME properties (see Table 5 below) represented a highly attractive lead. To further optimise *Leishmania* CRK3 potency, we synthesised an array of analogues by modifying the phenyl ring (Scheme 3). The activities of compounds **50**, **52**–**61** are summarised in Table 4.

**Table 4:** *Leishmania* CRK3–CYC6 and *Hs*CDK2–CYCA activity for the urea series

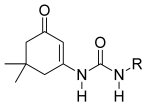			
Compd[a]	R	CRK3–CYC6 IC_50_ [μm][b]	*Hs*CDK2–CYCA IC_50_ [μm][c]
**50**	phenyl	0.095	>100
**52**	methyl	>100	>100
**53**	cyclohexylmethyl	3.9	>100
**54**	cyclohexyl	0.098	>100
**55**	2-methoxyphenyl	8.0	>100
**56**	3-methoxyphenyl	0.04	>100
**57**	4-methoxyphenyl	0.89	>100
**58**	4-benzyloxyphenyl	0.11	>100
**59**	4-benzoic acid methyl ester	3.3	>100
**60**	4-hydroxyphenyl	3.3	5.9
**61**	phenyl	>100	>100

[a] Compounds **50** and **52**–**60**: 3-(5,5-dimethylcyclohex-2,3-ene)-1-one; compound **61**: 3-amino-cyclohex-2-eneone.

[b] Concentration required to inhibit *Leishmania* CRK3–CYC6 activity by 50 %; data represent the mean of two or more experiments.

[c] Concentration required to inhibit *Hs*CDK2–CYCA activity by 50 %; data represent the mean of two or more experiments.

**Table 5:** Properties of *Leishmania* CRK3–CYC6 inhibitors.[a]

Compd	*M*_r_ [Da]	log *P*[b]	log *D*[c]	HBD[d]	HBA[e]	PSA[f]	HIA[g]
**11**	306	4.5	4.5	2	4	58	+
**46**	348	4.6	4.6	1	5	64	+
**50**	258	3.2	3.2	2	4	58	+
**56**	340	3.1	3.1	2	5	67	+

[a] Properties were calculated using the StarDrop software package (http://www.optibrium.com/stardrop.php).

[b] Partition coefficient.

[c] Partition coefficient at pH 7.2.

[d] Number of hydrogen bond donors.

[e] Number of hydrogen bond acceptors.

[f] Polar surface area.

[g] Prediction of human intestinal absorption sufficient for an orally dosed drug.

##### Chemistry

The ureas **50** and **52**–**61** were made by direct reaction of isocyanates with amine **51** under elevated temperature.

##### Enzyme activity

The requirement for a substantial lipophilic group was demonstrated by compound **52** (R=methyl), which was inactive, whereas the cyclohexylmethylene derivative **53** regained some activity. Replacements of the phenyl ring of **50** with cyclohexyl **54** or substitution of the aromatic ring at the 3- or 4-positions (**56**–**58**) were well tolerated overall, but did not show a significant improvement in potency. However, substitution at the 2-position (compound **55**) or polar groups at the 4-position, such as ester **59**, resulted in up to a 100-fold loss of activity. The majority of analogues demonstrated excellent selectivity over *Hs*CDK2, whereas compound **60**, with a 4-hydroxy group on the R^2^ phenyl ring, was unselective possibly due to favourable interactions with the water pocket near Asp 45 in *Hs*CDK2. Modification of the cyclohexenyl moiety by removal of the *gem*-dimethyl groups (compound **61**) resulted in a complete loss of activity.

##### Kinase selectivity

Compounds **58** and **59** proved to be highly selective against a panel of 76 human kinases, with only **59** showing >50 % inhibition at 10 μm against one kinase, ERK8.

### Antiparasitic activity

Key compounds from each series were assayed against *L. major* wild-type promastigotes. Cell culture assays were performed as described recently.[11] Only compound **25** from series 7 translated into moderate cell activity; the other compounds showed no activity up to 50 μm. Due to close sequence identity across the kinetoplastid (*Leishmania* and *Trypanosoma* spp.) CRK3s, it was believed that these inhibitors may be useful against other trypanosomatid parasites. Selected examples from series 3, 5, 7, and 8 were tested for antiproliferative activity against *T. brucei brucei* grown in culture. Unfortunately, the activity against *Leishmania* CRK3–CYC6 did not translate into cellular activity.

The reason for the lack of translation of *Leishmania* CRK3 inhibition into antiparasitic activity is unknown and cannot be fully investigated, as assays to determine *Leishmania* CRK3 activity in situ within parasites are not available. Unfortunately, the lack of understanding of the signalling cascades within these parasites means the downstream substrates of *Leishmania* CRK3 are unknown, preventing the development of such assays. The compounds identified across the main series studied (Table 5) have physicochemical properties regarded as suitable for membrane penetration, so an antiproliferative effect against parasites would be expected to accompany inhibition of *Leishmania* CRK3. Possible explanations for the lack of activity could be: 1) the compounds are excluded from the cells by an efflux mechanism; 2) *Leishmania* CRK3 binds to a cyclin other than CYC6 in the cell, forming an alternative complex for which these compounds have much lower affinity; and 3) *Leishmania* CRK3 is not amenable to chemical inhibition, as it is either not essential due to bypass mechanisms, or a sufficiently high level of target inhibition cannot be achieved chemically to observe a strong antiproliferative effect.

## Conclusions

Genetic manipulation studies have suggested that *Leishmania* CRK3 kinase is essential; however, limited chemical validation has been carried out. Screening of a focussed set of kinase inhibitors against *Leishmania* CRK3–CYC6 was used to identify tool compounds suitable for chemical validation of this target. The screen identified a number of series as starting points for medicinal chemistry investigations. Optimisation of four of the series resulted in the identification of compounds with significant levels of activity against *Leishmania* CRK3–CYC6 with suitable selectivity against the highly homologous related human kinase CDK2 for two series. Classical SAR studies allowed the generation of very potent (nanomolar range) triazolopyridine inhibitors as a novel series of potent and selective inhibitors of *Leishmania* CRK3–CYC6 (IC_50_<5 nm) with >10 000-fold selectivity over the human homologue *Hs*CDK2. This is the first report of low-nanomolar species-selective *Leishmania* CRK3 inhibitors. Unfortunately, no *Leishmania* CRK3 inhibitors from these three series demonstrated significant activity against cell cultures of the *L. major* parasite, indicating that the enzyme is not essential for parasite proliferation. In addition, the lack of antiparasitic activity of these potent inhibitors of *Leishmania* CRK3 suggests that genetic studies alone are insufficient to prove validity of *Leishmania* CRK3 as a drug target; chemical validation is also required.

## Experimental Section

### Preparation of *Leishmania* CRK3:CYC6

Briefly, *L. major* CYC6 (LmjF32.3320; http://www.genedb.org) was cloned into pET15b to give plasmid pGL1218; this provides an N-terminal histidine tag. The *L. major* and *L. mexicana* CRK3 proteins are identical, apart from a single Thr for Ala substitution at position 217. *L. mexicana* CRK3 was therefore used as a generic *Leishmania* CRK3. *L. mexicana* CRK3 was cloned into pET28a to give pGL751a. *E. coli* BL21 (DE3) pLys-S cells were transformed with plasmid pGL1218 and then pGL751a to provide a CRK3–CYC6 co-expression cell line. Cell culture (1 L volume) was induced with 1 mm IPTG at 19 °C overnight with agitation. After 16 h, cells were harvested by centrifugation at 4000 *g* for 15 min and resuspended in ice-cold PBS (pH 7.4) supplemented with DNAse 1 (10 μg mL^−1^; Invitrogen) and lysozyme (100 μg mL^−1^; Sigma) for 60 min on ice. The cell lysate was sonicated (four cycles of 30 s on/30 s off). The cell lysate was harvested by centrifugation at 12 000 *g* for 20 min, and the soluble extract was filtered through a 0.2 μm filter syringe. The proteins were purified by BioCAD chromatography using a metal chelate Ni^2+^-charged column followed by a Hiload 16/60 Superdex-200 gel filtration column. The eluted CRK3–CYC6 complex was stored in buffer (20 mm HEPES pH 7.4, 50 mm NaCl, 2 mm EGTA, 2 mm dithiothreitol (DTT), and 0.02 % Brij-35) with 10 % glycerol at −80 °C.

### CDK2

Recombinant full-length human CDK2 protein was expressed in an *E. coli* expression system and contained an N-terminal glutathione S-transferase (GST) tag. Human cyclin A2 (S171–L432) was also expressed in an *E. coli* expression system and contained an N-terminal GST tag. Both proteins were purified on a glutathione (GSH) Sepharose column, the tags were cleaved, and the cleaved proteins were purified by gel filtration chromatography (30.4 and 34.1 kDa, respectively). The CDK2–cyclin A2 complex was activated by using active CAK1.

### Enzyme assays

IMAP-FP Screening Express Kits and the carboxyfluorescein (5FAM)-labelled peptide substrate (5FAM-GGGRSPGRRRRK-OH) were purchased from Molecular Devices. [γ-^33^P]ATP (cat. # NEG602H) and streptavidin-coated FlashPlate PLUS 96-well plates (cat. # SMP103A) were purchased from PerkinElmer, whilst the custom-made biotinylated peptide substrate (biotin-PKTPKKAKKL-OH) used in the radiometric assay was supplied by Pepceuticals Ltd. All other reagents were of analytical grade.

### CRK3 screen

The focussed kinase library of compounds was solubilised in DMSO with a maximum final assay DMSO concentration of 1 % (*v*/*v*). The initial compound library screen was conducted using an immobilised metal-ion affinity partitioning (IMAP) assay platform with fluorescence polarisation detection (IMAP-FP) and a 5FAM-labelled peptide substrate. Assays were carried out in black polystyrene 384-well plates at a final assay volume of 20 μL per well comprising IMAP reaction buffer containing 1 mm DTT, 30 μm test compound, 0.1 μm peptide substrate, 100 μm ATP, and 1 nm CRK3–CYC6 enzyme complex. Reaction was initiated by the addition of enzyme and incubation continued for 60 min at 20 °C before termination by the addition of 50 μL IMAP binding solution. Plates were left to equilibrate at 20 °C for a further 30–60 min prior to reading fluorescence polarisation (*λ*_ex_ 480 nm/P/S, *λ*_em_ 535 nm) on an EnVision 2102 multilabel plate reader (PerkinElmer).

### Radiometric assays

*CRK3 and CDK2:* A radiometric assay was adopted for hit follow-up and all subsequent compound potency determinations. CRK3 assays were carried out in polypropylene 96-well plates at a final assay volume of 50 μL per well comprising 50 mm Tris-HCl buffer (pH 7.5), 10 mm magnesium acetate, 0.2 mm EGTA, 2 mm DTT, 1 mg mL^−1^ bovine serum albumin, 0.02 % *v*/*v* Brij-35, test compound, 1 μm peptide substrate, 1 μm ATP comprising 7.4 kBq per well [γ-^33^P]ATP, and 5 nm CRK3–CYC6 enzyme complex. Reaction was initiated by the addition of the ATP/substrate cocktail, and plates were incubated for 60 min at 20 °C on a plate shaker before reaction termination by the addition of 50 mm EDTA at 50 μL per well. An aliquot of 90 μL per well was transferred to 96-well streptavidin-coated FlashPlates, which were then allowed to equilibrate on a plate shaker for 20 min prior to washing with PBS containing 0.1 % *v*/*v* Tween-20 (2×250 μL per well). Plates were sealed and transferred to a TopCount NXT HTS scintillation counter (PerkinElmer) to quantify ^33^P incorporation. The counter-screen CDK2 assays were also carried out in polypropylene 96-well plates at a final assay volume of 50 μL per well comprising 50 mm Tris-HCl (pH 7.5) containing 100 mm NaCl, 10 mm magnesium acetate, 0.2 mm EGTA, 2 mm DTT, 0.02 % *v*/*v* Brij-35, test compound, 1 μm peptide substrate, 1 μm ATP comprising 7.4 kBq per well [γ-^33^P]ATP, and 2 nm CDK2–CYCA enzyme complex. Reaction was initiated by the addition of the ATP/substrate cocktail, and plates were incubated for 60 min at 20 °C on a plate shaker before reaction termination by the addition of 50 mm EDTA at 50 μL per well. FlashPlate transfer, washing, and counting was as described above for CRK3. In both primary and secondary assays, maximal signal (*control*) was determined in the absence of inhibitors, whilst basal signal was determined in the absence of enzyme. All assay plates included staurosporine as a reference inhibitor.

### Data analysis

IMAP-FP output: fluorescence polarisation data were expressed in millipolarisation (mP) units, defined as: mP=1000×[(*S*−*G*×*P*)/(*S*+*G*×*P*)], for which *S* (S-plane)=parallel-polarised light, *P* (P-plane)=perpendicular-polarised light, and *G* (grating)=instrument- and assay-dependent factor.

For all compounds in the primary screen, data were normalised to the average of the maximum uninhibited response (CTRL) as follows [Eq. (1)]:


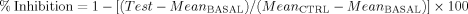
(1)

The quality of the primary screening assay was determined by calculating the *Z′* value as follows [Eq. (2)]:


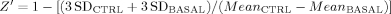
(2)

For which SD_CTRL_ and SD_BASAL_ represent the standard deviations of the response from 8–12 control wells (uninhibited signal) and 8–12 basal signal wells, respectively.

### IC_50_ analysis

Data were normalised to percent inhibition and concentration–response curves plotted using a four-parameter logistic fit equation (model 205) in an ActivityBase (IDBS) template using XLfit.

### Molecular modelling

*Homology modelling:* CRK3 target sequences from *T. brucei* and *L. mexicana* were aligned with the CDK2 sequence using T-coffee.[23] Subsequently, Modeller 9.2[24] was used to build homology models of CRK3, whereas the CDK2 crystal structures 1PYE[19] and 1H1P[24] were used as templates. Modeller was run with default settings, and only the highest-scoring structure was used to generate ligand binding modes.

*Generation of putative binding modes:* Relibase+[25] was used to search for CDK2 crystal structures co-crystallised with ligands similar to the selected hit series compounds. By superimposing the binding site of the CRK3 homology model onto the binding site of the relevant crystal structure, an initial binding mode with CRK3 was generated. Using MOLOC, (Gerber Molecular Design), the crystal structure ligand was modified to obtain the desired CRK3 inhibitor, and its position was optimised with the MAB force field.[26] To allow for uncertainties in the homology model, the amino acid side chains facing the binding sit were kept flexible during minimisation.

### Chemistry

^1^H NMR spectra were recorded on a Bruker Avance DPX 500 instrument unless otherwise stated. Chemical shifts (*δ*) are expressed in ppm. Signal splitting patterns are described as singlet (s), broad singlet (bs), doublet (d), triplet (t), quartet (q), multiplet (m), or combination thereof. Low-resolution electrospray (ES) mass spectra were recorded on a Bruker MicroTof mass spectrometer, run in positive ion mode, using either MeOH, MeOH/H_2_O (95:5), or H_2_O/CH_3_CN (1:1)+0.1 % formic acid as the mobile phase. High-resolution electrospray MS measurements were performed on a Bruker MicroTof mass spectrometer. LC–MS analyses were performed with an Agilent HPLC 1100 (Phenomenex Gemini Column 5 μ C_18_ 110A 50×3.0 mm, eluting with 20 % MeOH/H_2_O, 0–3 min) and a diode array detector in series with a Bruker MicroTof mass spectrometer. All synthesised compounds were determined to be >95 % pure by LC–MS. Thin-layer chromatography (TLC) was carried out on Merck silica gel 60 F_254_ plates using UV light and or KMnO_4_ for visualisation. TLC data are given as the *R*_f_ value with the corresponding eluent system specified in brackets. Column chromatography was performed using RediSep 4 or 12 g silica pre-packed columns. All reactions were carried out under dry and inert conditions, unless otherwise stated. Additional experimental procedures can be found in the Supporting Information.

**3-(4-Chlorophenyl)-3-oxopropanenitrile (12)**: CH_3_CN (1.5 mL, 29.4 mmol) was dissolved in dry THF (80 mL) under N_2_. The reaction mixture was cooled to −78 °C, and LiN(SiMe_3_)_2_ (30 mL of a 1 m solution in toluene, 30 mmol) was added slowly so that the temperature was kept below −60 °C. The reaction mixture was stirred for 1 h at −78 °C, and then methyl 4-chlorobenzoate (5 g, 29.3 mmol) was added in one portion. The reaction was allowed to warm slowly to RT and stirred for 16 h before quenching with saturated aqueous NH_4_Cl (100 mL). The solution was further diluted with H_2_O (100 mL) and EtOAc (200 mL). The resulting precipitate was collected by filtration and washed with Et_2_O (100 mL) to give **12** as a white solid (3.39 g, 18.9 mmol, 64 %). ^1^H NMR ([D_6_]DMSO, 500 mhz): *δ*=7.66 (1 H, s, ArH), 7.34 (1 H, s, ArH), 6.71 (2 H, s, ArH), 3.35 ppm (2 H, s, CH_2_); LC–MS (ES) [*M*+H], 100 %.

**5-(4-Chlorophenyl)-1*H*-pyrazol-3-amine (13)**: **12** (15.9 g, 93.3 mmol) was dissolved in EtOH (250 mL), N_2_H_4_⋅H_2_O (5 mL, 102.6 mmol) was added, and the reaction was heated at reflux with stirring for 24 h. The reaction was then cooled to RT and concentrated in vacuo to give a yellow oil. Trituration with CH_2_Cl_2_ gave a white solid **13** (14.8 g, 76 mmol, 86 % yield). ^1^H NMR (CDCl_3_, 500 mhz): *δ*=9.15 (1 H, bs, NH), 7.42–7.40 (2 H, m, ArH), 7.34–7.32 (2 H, m, ArH), 5.85 (1 H, s, ArH), 3.76 ppm (2 H, s, NH_2_); LC–MS (ES) [*M*+H], 100 %.

### General method for the acylation of 3-aminopyrazoles

***N*****-[5-(4-Chlorophenyl)-1*H*-pyrazol-3-yl]benzamide (8)**: **13** (97 mg, 0.5 mmol), DIPEA (90 μL, 0.6 mmol), and benzoyl chloride (64 μL, 0.55 mmol) were dissolved in dry 1,4-dioxane (2 mL) in a microwave reaction tube, capped and heated at 150 °C for 5 min in a microwave reactor. The reaction was then cooled to RT and PS isocyanate (0.25 g resin, 1.44 mmol g^−1^ loading) was added. The reaction tube was re-capped and heated at 160 °C for 5 min in the microwave reactor. The reaction was cooled, filtered to remove the resin, which was washed with 1,4-dioxane (2 mL). The reaction mixture was concentrated in vacuo, then partitioned between Et_2_O (3 mL) and NaOH (3 mL of a 2 m solution in H_2_O), and the mixture was stirred at RT for 1 h. The resulting solid was collected by filtration and washed with H_2_O (2 mL) and Et_2_O (2×2 mL) to give **20** (38 mg, 0.13 mmol, 26 %). ^1^H NMR ([D_6_]DMSO, 500 mhz): *δ*=8.05–8.03 (2 H, m, ArH), 7.80–7.79 (2 H, m, ArH), 7.53–7.48 (5 H, m, ArH), 6.86 ppm (1 H, s, ArH); LC–MS (ES) [*M*+H] (100 %).

***N*****-[5-(4-Chlorophenyl)-1*H*-pyrazol-3-yl]cyclohexanecarboxamide (11)**: Prepared by following the same procedure as **8** to afford **11** (63 mg, 42 %) as a white solid. ^1^H NMR ([D_6_]DMSO, 500 mhz): *δ*=12.92 (1 H, s, NH), 10.54 (1 H, s, NH), 7.75–7.73 (2 H, m, ArH), 7.50–7.49 (2 H, m, ArH), 6.82 (1 H, s, ArH), 2.39–2.33 (1 H, m, CH), 1.78–1.64 (5 H, m, CH), 1.41–1.10 ppm (5 H, m, CH); LC–MS (ES) [*M*+H], (97 %).

***N*****-(6-Bromo[1,2,4]triazolo[1,5-*a*]pyridinyl)cyclohexanecarboxamide (18)**: A mixture of triazolopyridine **16** (1.49 g, 7.0 mmol) and DMAP (85 mg, 0.7 mmol, 10 mol %) in pyridine (anhydrous, 30 mL) was cooled to 0 °C under argon, cyclohexylcarbonyl chloride (0.94 mL, 1.03 g, 7.0 mmol) was added dropwise, and the mixture was warmed to RT and stirred for 17 h. It was then concentrated in vacuo, and the crude mixture was partitioned between Et_2_O (20 mL) and H_2_O (10 mL), extracted with Et_2_O (2×10 mL), and the organic layer (suspension) separated from the aqueous layer and was passed directly through a filter funnel. The precipitate was collected and dried in vacuo to afford pure cyclohexylamide **18** (1.87 g, 83 %) as a colourless solid. ^1^H NMR (500 mhz, CDCl_3_): *δ*=10.76 (1 H, bs, NH), 9.27 (1 H, dd, ArH, *J*=1.9 and 0.7 Hz), 7.77 (1 H, dd, ArH, *J*=9.4 and 1.9 Hz), 7.65 (1 H, dd, ArH, *J*=9.4 and 0.7 Hz), 1.80 (2 H, d, cyclohexyl-H, *J*=12.6 Hz), 1.74 (2 H, dd, cyclohexyl-H, *J*=9.6 and 3.1 Hz), 1.64 (1 H, d, cyclohexyl-H, *J*=11.1 Hz), 1.38 (2 H, dd, cyclohexyl-H, *J*=12.2 and 2.7 Hz), 1.17–1.30 ppm (4 H, m, cyclohexyl-H); LC–MS [ES+]: *m*/*z* 395 and 397 (^79^Br [*M*+H] and ^81^Br [*M*+H]^+^) *t*_R_=0.9–1.1 min.

*General procedure A:* Suzuki reaction on the triazolopyridine core (**18** to **19**). Compound **4** (1 equiv), *N*,*N*-dimethylformamide (DMF; 4 mL (mmol **4**)^−1^, saturated aqueous K_2_CO_3_ (4 mL mmol^−1^), Pd(PPh_3_)_4_ (1 %), and boronic acid (2 equiv) were heated in a microwave reactor for 10 min at 140 °C, diluted with EtOAc, extracted and washed with LiCl (5 %, 1×10 mL), H_2_O (3×10 mL), and brine (1×10 mL), dried over MgSO_4_, filtered, and the solvent was removed in vacuo. Column chromatography was performed by eluting with petroleum ether (40–60): ether afforded the desired aryl-substituted triazolopyridines.

*General procedure B:* Amide coupling on the aryl-substituted triazolopyridine core (**20** to **19**). Triazolopyridine **16** (1 equiv), DMAP (10 mol %), and pyridine (anhydrous, 7 mL mmol^−1^) was cooled to 0 °C under argon, and the acid chloride (1 equiv) was added dropwise. The mixture was warmed to RT and stirred for 17 h, concentrated in vacuo, and the crude mixture partitioned between Et_2_O (20 mL) and H_2_O (10 mL), was extracted with Et_2_O (2×10 mL), and the organic layer (suspension) separated from the aqueous layer; this was passed directly through a filter funnel, and the white precipitate was collected to afford the desired product.

***N*****-{6-(3-Methylphenyl)-[1,2,4]triazolo[1,5-*a*]pyridinyl}cyclohexanecarboxamide (25)**: Prepared according to general procedure A on a 0.15 mmol scale to afford the product as a colourless solid (36 mg, 72 %). ^1^H NMR (500 mhz, CDCl_3_): *δ*=8.77 (1 H, s, ArH), 8.47 (1 H, bs, NH), 7.78 (1 H, dd, ArH, *J*=9.2 and 1.8 Hz), 7.65 (1 H, d, ArH, *J*=9.2 Hz), 7.36–7.41 (3 H, m, ArH), 7.23 (1 H, d, ArH, *J*=7.4 Hz), 2.47 (3 H, s, CH_3_), 2.05–2.02 (2 H, m, cyclohexyl-H), 1.86–1.88 (2 H, m, cyclohexyl-H), 1.70–1.75 (1 H, m, cyclohexyl-H), 1.63–1.60 (2 H, m, cyclohexyl-H), 1.27–1.31 ppm (4 H, m, cyclohexyl-H); LC–MS [ES+]: *m*/*z* 335 [*M*+H]^+^ *t*_R_=3.6–3.7 min.

***N*****-{7-(3,5-Dimethylphenyl)-[1,2,4]triazolo[1,5-*a*]pyridinyl}cyclohexanecarboxamide (33)**: Prepared according to general procedure A on a 0.46 mmol scale. Purification by column chromatography eluting with Et_2_O afforded the product as a colourless solid (34 mg, 21 %). ^1^H NMR (500 mhz, DMSO): *δ*=10.72 (1 H, s, NH), 9.15 (1 H, s, ArH), 7.97 (1 H, dd, ArH, *J*=9.2 and 1.8 Hz), 7.74 (1 H, dd, ArH, *J*=9.2 and 0.7 Hz), 7.42 (2 H, s, ArH), 7.07 (1 H, s, ArH), 2.53–2.51 (1 H, m, CH), 2.37 (6 H, s, CH_3_), 1.84–1.75 (4 H, m, CH), 1.68–1.65 (1 H, m, CH), 1.45–1.39 (2 H, m, CH), 1.32–1.19 ppm (3 H, m, CH); LC–MS [ES+]: *m*/*z* 349 [*M*+H]^+^ *t*_R_=4.8–5.0 min.

**2-Cyclopentyl-*N*-(6-*m*-tolyl-[1,2,4]triazolo[1,5-*a*]pyridinyl)acetamide (44)**: Prepared by following general procedure B on a 0.22 mmol scale to afford the desired product (20 mg, 27 %). ^1^H NMR (500 mhz, CDCl_3_): *δ*=8.78 (1 H, bs, NH), 7.79 (1 H, dd, ArH, *J*=9.2 and 1.8 Hz), 7.66 (1 H, dd, ArH, *J*=9.2 and 0.7 Hz), 7.42 (1 H, d, ArH, *J*=7.5 Hz), 7.37–7.40 (2 H, m, ArH), 2.47 (3 H, s, ArH), 2.37–2.44 (1 H, m, CH), 1.92–1.99 (2 H, m, CH_2_), 1.63–1.72 (2 H, m, cyclopentyl-H), 1.59–1.62 (4 H, m, cyclopentyl-H), 1.23–1.31 ppm (2 H, m, cyclopentyl-H); LC–MS [ES+]: *m*/*z* 335 [*M*+H]^+^ *t*_R_=3.7–3.8 min.

**1-Cyclohexyl-3-(5,5-dimethyl-3-oxocyclohex-1-en-1-yl)urea (54)**: Prepared by following general procedure A to afford **54** as a white solid (47 mg, 25 % yield). ^1^H NMR (DMSO, 500 mhz): *δ*=8.32 (1 H, s, NH), 6.42 (1 H, d, *J*=7.6 *Hz*, NH), 6.40 (1 H, s, CH), 3.43–3.41 (1 H, m, CH), 2.23 (2 H, s, CH_2_), 2.05 (2 H, s, CH_2_), 1.78–1.75 (2 H, m, CH), 1.65–1.63 (2 H, m, CH), 1.54–1.51 (1 H, m, CH), 1.33–1.26 (2 H, m, CH), 1.21–1.11 ppm (3 H, m, CH) and 0.99 (6 H, s, Me); LC–MS 97 % ([*M*+H], 265).

**1-(5,5-Dimethyl-3-oxocyclohex-1-en-1-yl)-3-(3-methoxyphenyl)urea (56)**: Prepared by following general procedure A to afford **56** as a white solid (98 mg, 47 % yield). ^1^H NMR (DMSO, 500 mhz): *δ*=8.92 (1 H, s, NH), 8.65 (1 H, s, NH), 7.22–7.12 (2 H, m, ArH), 6.92–6.6.91 (1 H, m, ArH), 6.62–6.60 (1 H, m, CH), 6.43 (1 H, s, CH), 3.73 (3 H, s, Me), 2.33 (2 H, s, CH_2_), 2.11 (2 H, s, CH_2_) and 1.02 ppm (6 H, s, Me); LC–MS 99 % ([*M*+H], 289).

## Supplementary material

Detailed facts of importance to specialist readers are published as ”Supporting Information”. Such documents are peer-reviewed, but not copy-edited or typeset. They are made available as submitted by the authors.
