# Supplementary material for: Identification of Inhibitors of the Leishmania cdc2-Related Protein Kinase CRK3
Source: ChemMedChem. 2011 Sep 13;6(12):2214–24. doi: 10.1002/cmdc.201100344 (PMC3272345; doi:10.1002/cmdc.201100344)
Supplement: Supplementary file 1 [file cmdc0006-2214-SD1.pdf]

CHEMMEDCHEM
Chemistry & Drug Discovery

Supporting Information

© Copyright Wiley-VCH Verlag GmbH & Co. KGaA, 69451 Weinheim, 2011

Identification of Inhibitors of the *Leishmania* cdc2-Related Protein Kinase CRK3

Laura A. T. Cleghorn,[a] Andrew Woodland,[a] Iain T. Collie,[a] Leah S. Torrie,[a] Neil Norcross,[a] Torsten Luksch,[a] Chido Mpamhanga,[a] Roderick G. Walker,[b] Jeremy C. Mottram,[b] Ruth Brenk,[a] Julie A. Frearson,[a] Ian H. Gilbert,[a] and Paul G. Wyatt*[a]

cmdc_201100344_sm_miscellaneous_information.pdf

Suppoting Information

Table of contents.

Table S1: Sequence identity between CDK2 and CRK3s from kinetoplastid parasites.

Figure S1. Average potency from IMAP-FP platform for 24 of the screening hits identified.

Table S2. *Leishmania* CRK3:CYC6 and *Hs*CDK2:CDK2 activity for series 3.

Table S3. *Leishmania* CRK3:CYC6 and *Hs*CDK2/cyclinA activity for series 5.

Table S4: Kinase selectivity data for compound series 3.

Table S5: Kinase selectivity data for compound series 5.

Table S6: Kinase selectivity data for compound series 7.

Table S7: Metabolic turnover of **20** and **25**

**Table S1**. Sequence identity between CDK2 and CRK3s from kinetoplastid parasites (all sequences are compared to *T. brucei* CRK3)			
SWISS_PROT Entry Name	Protein Name	Organism	Sequence Identity
Q38BA2_9TRYP	Cell division related protein kinase 2, putative (EC 2.7.1.37).	*Trypanosoma brucei*	100.0
Q94796_TRYCR	Cdc2-related protein kinase 3	*Trypanosoma cruzi*	82.0
O15851_LEIME	Cdc2-related kinase 3	*Leishmania* mexicana	78.0
O96526_LEIMA	Cdc2-related kinase 3	*Leishmania* major	78.0
CDK2_HUMAN	Cell division protein kinase 2 (EC 2.7.11.22) (p33 protein kinase)	Homo sapiens	58.0

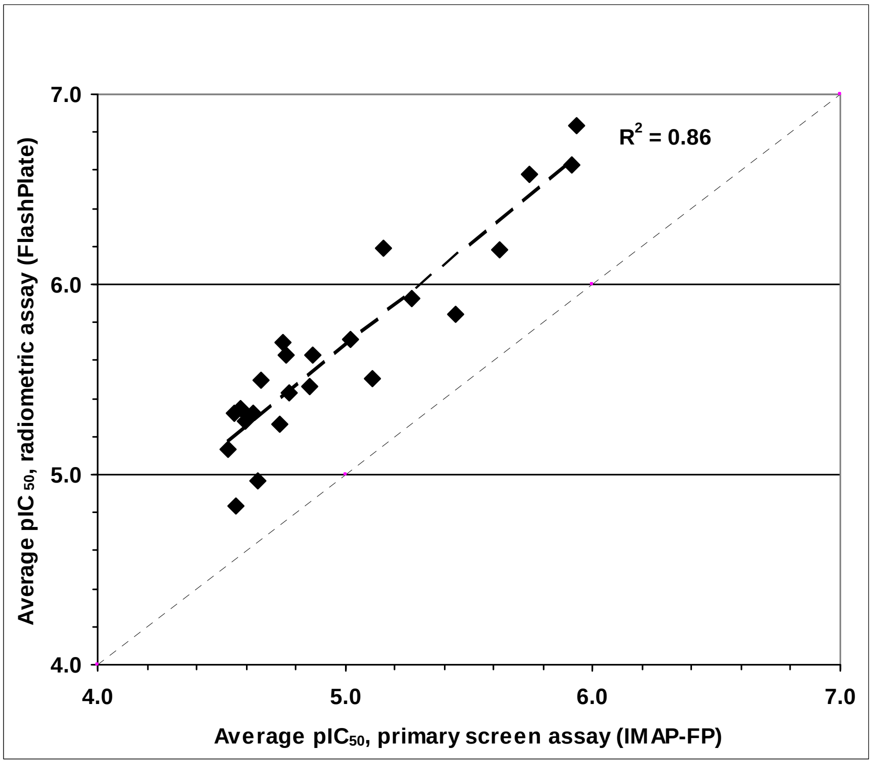

Figure S1. Average potency, expressed as pIC_{50}, as determined by IMAP-FP platform correlated to the potency value determined in the radiometric assay platform for 24 of the screening hits identified. Correlation coefficient (R2) is 0.86.

Table S2. *Leishmania* CRK3:CYC6 and *Hs*CDK2:CDK2 activity for series 3 pyrazolopyrimidines

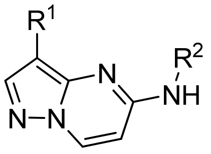

R^1	R^2	Leishmania CRK3:CYC6 %I at 30μM[a]	Leishmania CRK3:CYC6 IC_{50} (μM)[b]	Hs CDK2/CYCA IC_{50} (μM)[c]
H	3,4,5-trimethoxyphenyl	0	-	-
3,4,-dimethoxyphenyl	3,4,5-trimethoxyphenyl	72	6	-
3-chlorophenyl	3,4,5-trimethoxyphenyl	25	-	-
3-carboxamidophenyl	4-morpholinophenyl	93	0.26	0.43
3,4,5-trimethoxyphenyl	*N*,*N*-dimethylaminoethyl	3	-	-
3-pyridyl	4-morpholinophenyl	-	0.54	-
3-methylphenyl	4-morpholinophenyl	-	2.6	>100
Br	4-morpholinophenyl	-	10	-

[a] Percentage inhibition of *Leishmania* CRK3:CYC6 activity at 30 μM [b] Concentration required to inhibit CRK3:CYC6 activity by 50%: data represents the average of 2 or more experiments. [c] Concentration required to inhibit *Hs*CDK2/CYCA activity by 50%: data represents the average of 2 or more experiments.

**Table S3**. *Leishmania* CRK3:CYC6 activity and selectivity compared to *Hs*CDK2/cyclinA for pyrazoles			
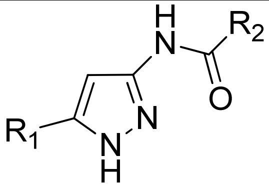			
R_1	R_2	*Leishmania* CRK3:CYC6 IC_{50} (μM)	*Hs* CDK2:cyclinA IC_{50} (μM)
Cyclopropyl	Cyclobutyl	2.1	0.089
2,6-Difluorophenyl	Cyclobutyl	1.3	1.9
3-pyridyl	Cyclobutyl	20	6.6
Benzyl	Cyclobutyl	11	1.1
Benzyl	Methyl	64	4.0
Benzyl	Phenyl	9.2	3.0
3-pyridyl	Phenyl	23	100
2,6-Difluorophenyl	Phenyl	3.0	100
2,6-Difluorophenyl	Methyl	9.0	3.4
Cyclopropyl	Phenyl	1.7	0.15
4-Chlorophenyl	Cyclohexyl	0.33	100
4-Chlorophenyl	3-Phenol	1.1	5.4
4-Chlorophenyl	2-Thiophene	1.2	3.5
4-Chlorophenyl	4-Methoxyphenol	33	nd[c]
4-Chlorophenyl	Cyclopropyl	1.5	nd[c]
4-Chlorophenyl	3-Fluorophenyl	100	nd[c]
4-Chlorophenyl	2-Methylphenyl	100	nd[c]

[a] Concentration required to inhibit *Leishmania* CRK3:CYC6 activity by 50%: data represents the average of 2 or more experiments. [b] Concentration required to inhibit *Hs*CDK2/cyclinA activity by 50%: data represents the average of 2 experiments. [c] nd = not determined

Table S4: Kinase selectivity data for compound series 3. Compounds were incubated against 76 recombinant human kinases at a concentration of 10 μM. The percentage activity of each kinase is shown. Where highlighted in red the enzymes have < 10 % or > 100 % activity; when highlighted in orange > 10 % but < 29 %; when highlighted in yellow > 30 % but < 49 %.

	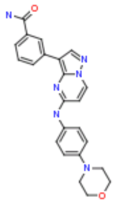 10μM	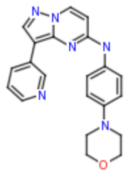 10μM
MKK1	**25**	**41**
ERK1	**93**	**90**
ERK2	**87**	**96**
JNK1	**80**	**76**
JNK2	**74**	**64**
p38a MAPK	**78**	**79**
P38b MAPK	**77**	**70**
p38g MAPK	**57**	**74**
p38s MAPK	**72**	**88**
ERK8	**5**	**8**
RSK1	**17**	**38**
RSK2	**37**	**52**
PDK1	**36**	**63**
PKBa	**67**	**84**
PKBb	**41**	**55**
SGK1	**86**	**61**
S6K1	**18**	**49**
PKA	**53**	**62**
ROCK 2	**57**	**55**
PRK2	**41**	**86**

PKCa	20	33
PKC zeta	27	61
PKD1	136	160
MSK1	18	44
MNK1	23	86
MNK2	25	54
MAPKAP-K2	93	95
PRAK	70	70
CAMKKb	42	42
CAMK1	54	68
SmMLCK	45	55
PHK	4	6
CHK1	59	29
CHK2	28	26
GSK3b	21	21
CDK2-Cyclin A	3	42
PLK1	51	53
PLK1 (Okadaic Acid)	49	53
AMPK	18	32
MARK3	17	64
BRSK2	33	40
MELK	28	10
CK1	59	46
CK2	12	20
DYRK1A	2	14
DYRK2	7	5
DYRK3	9	10
NEK2a	80	75
NEK6	177	133
IKKb	47	47

PIM1	27	18
PIM2	16	28
PIM3	4	9
SRPK1	48	61
MST2	15	44
EFK2	103	102
HIPK2	4	3
PAK4	66	50
PAK5	59	61
PAK6	76	71
Src	4	8
Lck	7	10
CSK	15	48
FGF-R1	11	5
IRR	4	12
EPH A2	65	69
MST4	73	77
SYK	80	66
YES1	9	12
IKKe	26	46
TBK1	17	58
IGF1-R	47	44
VEG-FR	15	8
BTK	9	38
IR-HIS	65	78
EPH-B3	29	33

**Table S5**: Kinase selectivity data for compound series 5. C Compounds were incubated against 76 recombinant human kinases at a concentration of 10 μM. The percentage activity of each kinase is shown. Where highlighted in red the enzymes have < 10 % or > 100 % activity; when highlighted in orange > 10 % but < 29 %; when highlighted in yellow > 30 % but < 49 %.	
	**Compound 11**
	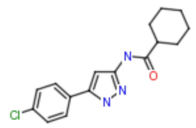
	**10μM**
MKK1	**68**
ERK1	**86**
ERK2	**91**
JNK1	**98**
JNK2	**87**
p38a MAPK	**84**
P38b MAPK	**66**
p38g MAPK	**79**
p38s MAPK	**83**
ERK8	**33**
RSK1	**76**
RSK2	**69**
PDK1	**99**
PKBa	**89**
PKBb	**75**
SGK1	**86**

S6K1	97
PKA	84
ROCK 2	88
PRK2	101
PKCa	103
PKC zeta	73
PKD1	184
MSK1	83
MNK1	82
MNK2	81
MAPKAP-K2	97
PRAK	76
CAMKKb	72
CAMK1	154
SmMLCK	89
PHK	75
CHK1	79
CHK2	92
GSK3b	27
CDK2-Cyclin A	71
PLK1	86
PLK1 (Okadaic Acid)	90
AMPK	86
MARK3	78
BRSK2	82
MELK	84
CK1	87
CK2	103
DYRK1A	65
DYRK2	84

DYRK3	87
NEK2a	90
NEK6	171
IKKb	100
PIM1	87
PIM2	81
PIM3	97
SRPK1	75
MST2	93
EFK2	105
HIPK2	89
PAK4	56
PAK5	67
PAK6	84
Src	105
Lck	73
CSK	81
FGF-R1	114
IRR	30
EPH A2	105
MST4	68
SYK	94
YES1	89
IKKe	72
TBK1	72
IGF1-R	99
VEG-FR	75
BTK	81
IR-HIS	86
EPH-B3	82

Table S6: Kinase selectivity data for compound series 7. Compounds were incubated against 76 recombinant human kinases at a concentration of 10 µM. The percentage activity of each kinase is shown. Where highlighted in red the enzymes have < 10 % or > 100 % activity; when highlighted in orange > 10 % but < 29 %; when highlighted in yellow > 30 % but < 49 %.

	**Compound 38**	**Compound 39**	**Compound 32**
	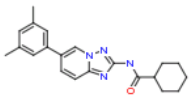	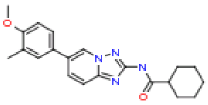	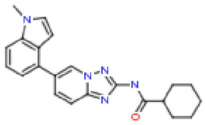
	**10µM**	**10µM**	**10µM**
MKK1	**47**	**43**	**59**
ERK1	**97**	**92**	**92**
ERK2	**93**	**90**	**86**
JNK1	**101**	**102**	**101**
JNK2	**83**	**82**	**94**
p38a MAPK	**104**	**95**	**95**
P38b MAPK	**91**	**87**	**79**
p38g MAPK	**102**	**93**	**88**
p38s MAPK	**86**	**75**	**74**
ERK8	**86**	**83**	**72**
RSK1	**62**	**70**	**68**
RSK2	**66**	**56**	**62**
PDK1	**96**	**103**	**96**
PKBa	**93**	**86**	**98**
PKBb	**67**	**66**	**59**
SGK1	**103**	**100**	**96**
S6K1	**95**	**107**	**105**
PKA	**84**	**82**	**82**
ROCK 2	**95**	**86**	**92**

PRK2	84	103	79
PKCa	87	97	95
PKC zeta	88	87	76
PKD1	92	110	80
MSK1	71	56	68
MNK1	80	84	79
MNK2	68	79	50
MAPKAP-K2	95	93	102
PRAK	65	60	63
CAMKKb	85	89	45
CAMK1	74	83	75
SmMLCK	92	88	85
PHK	72	69	50
CHK1	76	70	77
CHK2	99	93	76
GSK3b	70	73	78
CDK2-Cyclin A	105	98	90
PLK1	90	83	78
PLK1 (Okadaic Acid)	56	52	62
AMPK	93	97	98
MARK3	86	88	90
BRSK2	71	67	76
MELK	65	69	62
CK1	82	86	92
CK2	101	106	104
DYRK1A	59	36	15
DYRK2	62	48	11
DYRK3	59	33	4
NEK2a	89	83	77
NEK6	84	83	81

IKKb	95	97	93
PIM1	90	99	78
PIM2	103	100	91
PIM3	91	84	78
SRPK1	64	53	65
MST2	100	94	94
EFK2	107	107	102
HIPK2	114	102	86
PAK4	111	101	94
PAK5	88	74	68
PAK6	84	82	83
Src	98	93	89
Lck	71	80	79
CSK	84	81	68
FGF-R1	124	88	96
IRR	50	27	14
EPH A2	87	93	84
MST4	82	84	71
SYK	94	95	87
YES1	89	94	101
IKKe	100	108	104
TBK1	102	99	86
IGF1-R	78	94	104
VEG-FR	57	74	55
BTK	63	59	61
IR-HIS	80	78	77
EPH-B3	90	78	82

**Table S6 cont**: Kinase Selectivity Data compound series 7 Compounds were incubated against 76 recombinant human kinases at a concentration of 10 μM. The percentage activity of each kinase is shown. Where highlighted in red the enzymes have < 10 % or > 100 % activity; when highlighted in orange > 10 % but < 29 %; when highlighted in yellow > 30 % but < 49 %.		
	**Compound 66**	**Compound 25**
	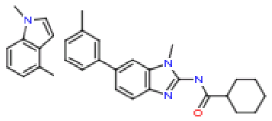	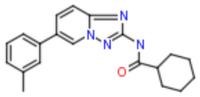
	**10μM**	**10μM**
MKK1	**67**	**74**
ERK1	**93**	**96**
ERK2	**99**	**75**
JNK1	**100**	**101**
JNK2	**86**	**82**
p38a MAPK	**96**	**86**
P38b MAPK	**78**	**82**
p38g MAPK	**109**	**92**
p38s MAPK	**81**	**78**
ERK8	**89**	**77**
RSK1	**85**	**67**
RSK2	**80**	**76**
PDK1	**97**	**101**
PKBa	**85**	**99**
PKBb	**78**	**75**
SGK1	**95**	**105**
S6K1	**112**	**86**
PKA	**84**	**87**
ROCK 2	**95**	**98**

PRK2	92	98
PKCa	138	110
PKC zeta	87	85
PKD1	84	89
MSK1	72	68
MNK1	104	82
MNK2	85	67
MAPKAP-K2	101	105
PRAK	74	75
CAMKKb	68	47
CAMK1	77	78
SmMLCK	79	83
PHK	71	56
CHK1	84	79
CHK2	76	95
GSK3b	71	71
CDK2-Cyclin A	106	101
PLK1	88	81
PLK1 (Okadaic Acid)	70	83
AMPK	102	99
MARK3	98	91
BRSK2	83	64
MELK	73	71
CK1	76	87
CK2	99	106
DYRK1A	59	10
DYRK2	45	24
DYRK3	13	19
NEK2a	90	95
NEK6	81	88

IKKb	103	90
PIM1	61	99
PIM2	108	100
PIM3	93	99
SRPK1	68	75
MST2	94	107
EFK2	106	105
HIPK2	108	96
PAK4	91	96
PAK5	71	82
PAK6	93	76
Src	88	88
Lck	90	77
CSK	87	94
FGF-R1	123	111
IRR	36	11
EPH A2	95	94
MST4	86	79
SYK	106	94
YES1	101	92
IKKe	95	86
TBK1	115	101
IGF1-R	112	97
VEG-FR	56	53
BTK	58	71
IR-HIS	78	115
EPH-B3	97	93

**Table S7:** Metabolic turnover of **20** and **25**							
Compd	Structure	Human % turnover at 40 min	Mouse % turnover at 30 min	PPB FU (%)	LogD	*T.brucei* EC_{50} µM	CDK2 IC_{50} µM
**25**	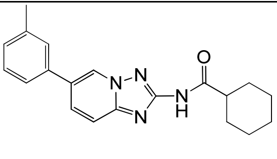	100	100	87	3.86	6.6	19
**20**	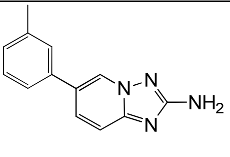	33	31	83	2.24	0.9	69

Assessed for stability in human and mouse liver microsomes, protein binding in mouse plasma and LogD determination. All assays were performed using the standard BioFocus DPI protocols.

Additional Experimental Section

***N*-(5-(4-Chlorophenyl)-1H-pyrazol-3-yl)acetamide (2)**. Prepared following the same procedure as **8** to afford **2** (2mg, 2%) as a white solid. ^{1}H NMR (d^6 DMSO, 500 MHz) δ 12.88 (1H, s, NH), 10.45 (1H, s, NH), 7.75-7.73 (2H, m, ArH), 7.52-7.50 (2H, m, ArH), 2.02 (3H, s, CH3); LCMS (ES) M+H (100%).

***N*-(5-(4-Chlorophenyl)-1*H*-pyrazol-3-yl)cyclobutanecarboxamide (7)**. Prepared following the same procedure as **8** to afford **7** (18 mg, 12%) as a white solid. ^{1}H NMR (d^6 DMSO, 500 MHz) δ 12.87 (1H, s, NH), 10.31 (1H, s, NH), 7.75-7.74 (2H, m, ArH), 7.52-7.50 (2H, m, ArH), 6.93 (1H, s, ArH), 3.25 (1H, qn, *J* = 8.4Hz, CH), 2.25-2.17 (2H, m, CH), 2.11-2.07 (2H, m, CH), 1.98-1.90 (1H, m, CH), 1.83-1.79 (1H, m, CH); LCMS (ES) M+H, (100%).

***N*-(5-(4-Chlorophenyl)-1*H*-pyrazol-3-yl)-2-phenylacetamide (9)**. Prepared following the same procedure as **8** to afford **9** (20mg, 13%) as a white solid. ^{1}H NMR (d^6 DMSO, 500 MHz) δ 7.73-7.72 (2H, m, ArH), 7.49-7.47 (2H, m, ArH), 7.34 (4H, broad s, ArH), 7.24 (1H, broad s, ArH), 6.80 (1H, s, ArH), 3.62 (2H, s, CH2); LCMS (ES) M+H, (100%).

***N*-(5-(4-Chlorophenyl)-1*H*-pyrazol-3-yl)nicotinamide (10)**. Prepared following the same procedure as **8** to afford **10** (28mg, 19%) as a light pink solid. ^{1}H NMR (d^6 DMSO, 500 MHz) δ 13.10 (1H, s, NH), 11.17 (1H, s, NH), 9.16-9.15 (1H, m, ArH), 8.76 (1H, s, ArH), 8.37-8.35 (1H, m, ArH), 7.81-7.80 (2H, m, ArH), 7.56-7.54 (3H, m, ArH), 7.11 (1H, s, ArH); LCMS (ES) M+H, (97%).

***N*-(6-(4-Hydroxy-3-methoxyphenyl) [1,2,4]triazolo[1,5a]pyridinyl)cyclohexanecarboxamide (14)**. Prepared according to general procedure A. ^{1}H NMR (500 MHz, DMSO): 10.67 (1H, s, NH), 9.25 (1H, s, OH), 9.16 (1H, s, ArH), 7.96 (1H, d, ArH, *J* = 8.9 Hz), 7.68-7.70 (1H, m, ArH), 7.35 (1H, s, ArH), 7.19-7.20 (1H, m, ArH), 6.87-6.89 (1H, m, ArH), 3.89 (3H, s, CH_3), 1.74-1.81 (4H, m, CH), 1.64-1.66 (1H, m, CH), 1.38-1.42 (2H, m, CH), 1.23-1.28 (3H, m, CH). LCMS [ES+]: *m/z* 367 $[M+H]^+$.

***N*-(6-Phenyl-[1,2,4]triazolo[1,5-a]pyridinyl)cyclohexanecarboxamide (22)**. Prepared according to general procedure B from [1,2,4]triazolo[1,5-a]pyridin-2-amine on a 0.12 mmol scale to afford the product as a colourless solid (20 mg, 54 %). ^{1}H NMR (500 MHz, DMSO) 10.73 (1H, s, NH), 9.22 (1H, bs, ArH), 8.00 (1H, dd, ArH, *J* = 9.2 and 1.8 Hz), 7.81 (2H, dd, ArH, *J* = 8.5 and 1.3 Hz), 7.76 (1H, dd, ArH, *J* = 9.3 and 0.6 Hz), 7.53 (2H, t, ArH, *J* = 7.4 Hz), 7.44 (1H, t, ArH, *J* = 7.4 Hz), 2.51-2.53 (1H, m, cyclohexyl-H), 1.83 (2H, bd, cyclohexyl-H, *J* = 12.7 Hz), 1.77 (2H, bd, cyclohexyl-H, *J* = 12.7 Hz), 1.66 (1H, bd, *J* = 11.6 Hz), 1.42 (2H, dq, cyclohexyl-H, *J* = 12.4 and 2.7 Hz,), 1.19-1.32 (3H, m, Cyclohexyl-H). LCMS [ES+]: *m/z* 321 $[M+H]^+$.

***N*-(6-(4-Hydroxyphenyl)-[1,2,4]triazolo[1,5-a]pyridinyl))cyclohexanecarboxamide (23)**. Prepared according to general procedure A on 0.62 mmol scale to afford the product as a colourless solid (63 mg, 30 %). ^{1}H NMR (500 MHz, DMSO): 10.69 (1H, s, NH), 9.73 (1H, s, OH), 9.07 (1H, bs, ArH), 7.91 (1H, dd, ArH, *J* = 9.3 and 1.8 Hz), 7.70 (1H, bd, ArH, *J* = 9.3 Hz), 7.62 (2H, dd, ArH, *J* = 6.6 and 2.0 Hz), 6.89 (2H, dd, ArH, *J* = 6.6 and 2.0 Hz), 2.51-2.53 (1H, m, cyclohexyl-H), 1.83 (2H, bd, Cyclohexyl-H, *J* = 12.9 Hz), 1.76 (2H, bd, cyclohexyl-H, *J* = 12.5 Hz), 1.66 (1H, bd, cyclohexyl-H, *J* = 11.2 Hz), 1.41 (2H, bq, Cyclohexyl-H, *J* = 12.1 Hz), 1.17-1.32 (3H, m, Cyclohexyl-H). LCMS [ES+]: *m/z* 337 $[M+H]^+$.

***N*-(6-(3-Methoxyphenyl)-[1,2,4]triazolo[1,5-a]pyridinyl)cyclohexanecarboxamide (24)**. Prepared according to general procedure A on a 0.44 mmol scale to afford the product as a colourless solid (84 mg, 52 %). ^{1}H NMR (500 MHz, DMSO): 10.73 (1H, s, NH), 9.26 (1H, bs, 2-H), 8.01 (1H, dd, ArH, *J* = 9.1, 1.8 Hz), 7.75 (1H, bd, ArH, *J* = 9.3 Hz), 7.44 (1H, t, ArH, *J* = 5.3 Hz), 7.37-7.38 (2H, m, ArH), 3.87 (3H, s, OCH_3), 7.00 (1H, dt, ArH, *J* = 7.2 and 1.6 Hz), 2.51-2.53 (1H, m, cyclohexyl-H), 1.83 (2H, bd, cyclohexyl-H, *J* = 13.0 Hz,), 1.77 (2H, bd, cyclohexyl-H, *J* = 12.8 Hz), 1.66 (1H, bd, *J* = 11.6 Hz), 1.42 (2H, bq, cyclohexyl-H, *J* = 12.0 Hz), 1.16-1.32 (3H, m, cyclohexyl-H). LCMS [ES+]: *m/z* 351 $[M+H]^+$.

***N*-(6-(3-Chlorophenyl) [1,2,4]triazolo[1,5a]pyridinyl)cyclohexanecarboxamide (26)**. Prepared according to general procedure A on a 0.15 mmol scale. Purification by column chromatography eluting with ether afforded the product as a colourless solid (21 mg, 38 %). ^{1}H NMR (500 MHz, $CDCl_3$): 8.74 (1H, s, ArH), 8.16 (1H, bs, NH), 7.72 (1H, dd, ArH, *J* = 9.1 and 1.8 Hz), 7.64 (1H, d, ArH, *J* = 9.1 Hz), 7.54 (1H, s, ArH), 7.40-7.44 (2H, m, ArH), 2.01 (2H, d, cyclohexyl-H, *J* = 11.4 Hz), 1.85 (2H, dt, cyclohexyl-H, *J* = 9.9 and 3.0 Hz), 1.71 (1H, d, cyclohexyl-H, *J* = 10.5 Hz), 1.26-1.39 (4H, m, cyclohexyl-H), 0.88-0.77 (2H, m, cyclohexyl-H). LCMS [ES+]: *m/z* 245 $[M+H]^+$ T_R = 2.9 min.

***N*-(6-(1-Napthyl) [1,2,4]triazolo[1,5a]pyridinyl)cyclohexanecarboxamide (27)**. Prepared according to general procedure A on a 0.15 mmol scale. Purification by column chromatography eluting with ether afforded the product as a colourless solid (30 mg, 53 %). ^{1}H NMR (500 MHz, $CDCl_3$): 8.81 (1H, s, ArH), 8.19 (1H, bs, NH), 7.95 (1H, s, ArH), 7.91 (1H, d, ArH, *J* = 8.6 Hz), 7.81-7.86 (3H, m, ArH), 7.63 (1H, d, ArH, *J* = 7.9 Hz), 7.60

(1H, dd, ArH, *J* = 7.3 and 1.9 Hz), 7.60 (1H, dd, ArH, *J* = 7.3 and 1.9 Hz), 7.45-7.51 (2H, m, ArH), 1.96 (2H, bd, cyclohex-H, *J* = 13.0 Hz), 1.78 (2H, dt, cyclohex-H, *J* = 13.0 and 3.3 Hz), 1.64 (1H, m, cyclohex-H, *J* = 10.6 Hz), 1.52-1.58 (1H, m, cyclohex-H), 1.18-1.34 (5H, m, cyclohex-H). LCMS [ES+]: *m/z* 371 $[M+H]^+$ T_R = 3.8 min.

***N*-(6-(4-Indole) [1,2,4]triazolo[1,5a]pyridinyl)cyclohexanecarboxamide (28)**. Prepared according to general procedure A on a 0.46 mmol scale. Purification by column chromatography eluting with ether afforded the product as a colourless solid (66 mg, 33 %). 1H NMR (500 MHz, DMSO δ): 10.74 (1H, s, NH), 11.42 (1H, s, NH), 9.02 (1H, s, ArH), 7.92 (1H, dd, ArH, *J* = 9.1 and 1.7 Hz), 7.80 (1H, d, ArH, *J* = 9.1 Hz), 7.51-7.50 (2H, m, ArH), 7.27-7.26 (2H, m, ArH), 6.62 (1H, s, ArH), 2.53-2.51 (1H, m, CH), 1.85 (2H, d, CH, *J* 12.5 Hz), 1.78 (2H, d, CH, *J* = 12.6 Hz), 1.68 (1H, d, CH, *J* = 12.0 Hz), 1.47-1.41 (2H, m, CH), 1.34-1.21 (3H, m, CH). LCMS [ES+]: *m/z* 360 $[M+H]^+$ T_R = 3.3-3.5 min.

***N*-(6-(4-N-Methylindole) [1,2,4]triazolo[1,5a]pyridinyl)cyclohexanecarboxamide (29)**. Prepared according to general procedure A on a 0.46 mmol scale. Purification by column chromatography eluting with ether afforded the product as a colourless solid (73 mg, 35 %).1H NMR (500 MHz, DMSO δ): 10.72 (1H, s, NH), 9.00 (1H, s, ArH), 7.95 (1H, dd, ArH, *J* = 9.1 and 1.7 Hz), 7.79 (1H, d, ArH, *J* = 9.1 Hz), 7.54 (1H, d, ArH, *J* = 8.0 Hz), 7.48 (1H, d, ArH, *J* = 3.1 Hz), 7.31 (1H, dd, ArH, *J* = 7.9 and 7.4 Hz), 7.25 (1H, d, ArH, *J* = 6.7 Hz), 6.59 (1H, d, ArH, *J* = 3.1 Hz), 3.87 (3H, s, CH_3), 2.53-2.51 (1H, m, CH), 1.84-1.82 (2H, m, CH), 1.77-1.75 (2H, m, CH), 1.67-1.65 (1H, m, CH), 1.45-1.38 (2H, m, CH), 1.31-1.11 (3H, m, CH). LCMS [ES+]: *m/z* 374 $[M+H]^+$ T_R = 3.5-3.7 min.

***N*-(7-(3-Morpholinophenyl) [1,2,4]triazolo[1,5a]pyridinyl)cyclohexanecarboxamide (30)**. Prepared according to general procedure A on a 0.46 mmol scale. Purification by column chromatography eluting with ether afforded the product as a colourless solid (68 mg, 36 %). 1H NMR (500 MHz, DMSO δ): 10.70 (1H, s, NH), 9.23 (1H, s, ArH), 7.99 (1H, d, ArH, *J* = 8.7 Hz), 7.72 (1H, d, ArH, *J* = 8.5 Hz), 7.37-7.32 (2H, m, ArH), 7.21 (1H, d, ArH, *J* = 6.4 Hz), 7.00 (1H, d, ArH, *J* = 7.7 Hz), 3.77 (4H, s, 2 x CH_2), 3.23 (4H, s, 2 x CH_2), 1.83-1.75 (4H, m, CH), 1.66-1.64 (1H, m, CH), 1.45-1.38 (2H, m, CH), 1.28-1.18 (3H, m, CH). LCMS [ES+]: *m/z* 406 $[M+H]^+$ T_R = 3.4-3.6 min.

***N*-(7-(2,6-Dimethylphenyl) [1,2,4]triazolo[1,5a]pyridinyl)cyclohexanecarboxamide (31)**. Prepared according to general procedure A on a 0.46 mmol scale. Purification by column chromatography eluting with ether afforded the product as a colourless solid (23 mg, 43 %). 1H NMR (500 MHz, CDCl3): 8.57 (1H, bs, NH), 8.39 (1H, s, ArH), 7.65 (1H, d, ArH, *J* = 9.0 Hz), 7.34 (1H, dd, ArH, *J* = 9.0 and 1.6 Hz), 7.25 (1H, d, ArH, *J* = 7.6 Hz), 7.16 (1H, d, ArH, *J* = 7.6 Hz), 2.07 (6H, s, 2 x CH_3), 2.01 (2H, d, cyclohexyl-H, *J* = 13.0 Hz), 1.83-1.86 (2H, m, cyclohexyl-H), 1.69-1.71 (1H, m, cyclohexyl-H), 1.56-1.61 (2H, m, cyclohexyl-H), 1.25-1.35 (4H, m, cyclohexyl-H). LCMS [ES+]: *m/z* 349 $[M+H]^+$ T_R = 3.7 min.

***N*-(7-(2,5-Dimethylphenyl) [1,2,4]triazolo[1,5a]pyridinyl)cyclohexanecarboxamide (32)**. Prepared according to general procedure A on a 0.46 mmol scale. Purification by column chromatography eluting with ether afforded the product as a colourless solid (55 mg, 34 %). 1H NMR (500 MHz, DMSO δ): 10.70 (1H, s, NH), 8.86 (1H, s, ArH), 7.72 (1H, d, ArH, *J* = 9.0 Hz), 7.64 (1H, dd, ArH, *J* = 9.0 and 1.7 Hz), 7.25 (1H, d, ArH, *J* = 8.4 Hz), 7.17-7.18 (2H, m, ArH), 2.53-2.51 (1H, m, CH), 2.34 (3H, s, CH_3), 2.25 (3H, s, CH_3), 1.83 (2H, d, CH, *J* = 13.0 Hz), 1.77 (2H, d, CH, *J* = 12.0 Hz), 1.66 (1H, d, CH, *J* = 11.5 Hz), 1.45-1.38 (2H, m, CH), 1.32-1.19 (3H, m, CH). LCMS [ES+]: *m/z* 349 $[M+H]^+$ T_R = 4.6-4.7 min.

***N*-(6-(4-Methoxy-3-methylphenyl) [1,2,4]triazolo[1,5a]pyridinyl)cyclohexanecarboxamide (34)**. Prepared according to general procedure A on a 0.46 mmol scale. Purification by column chromatography eluting with ether afforded the product as a colourless solid (67 mg, 33 %). 1H NMR (500 MHz, DMSO δ):10.69 (1H, s, NH), 9.12 (1H, s, ArH), 7.96 (1H, dd, ArH, *J* = 9.2 and 1.8 Hz), 7.72 (1H, d, ArH, *J* = 9.2 Hz), 7.63-7.61 (2H, m, ArH), 7.07 (1H, d, ArH, *J* = 8.7 Hz), 3.85 (3H, s, OCH_3), 2.53-2.51 (1H, m, CH), 2.25 (3H, s, CH_3), 1.84-1.81 (2H, m, CH), 1.75-1.78 (2H, m, CH), 1.68-1.65 (1H, m, CH), 1.42-1.41 (2H, m, CH), 1.29-1.22 (3H, m, CH). LCMS [ES+]: *m/z* 365 $[M+H]^+$ T_R = 3.6-3.8 min.

6-m-Tolyl-[1,2,4]triazolo[1,5-a]pyridine-3-amine (35). Prepared according to general procedure A on a 2.95 mmol scale. Purification by column chromatography eluting with ether afforded the product as a cream solid (660 mg, 100 %). ^{1}H NMR (500 MHz, $CDCl_3$): 8.48 (1H, d, ArH, *J* = 0.7 Hz), 8.10 (1H, d, ArH, *J* = 9.2 Hz), 7.80 (1H, dt, ArH, *J* = 9.2 and 1.7 Hz), 7.40 (1H, t, ArH, *J* = 7.6 Hz), 7.32-7.35 (2H, m, ArH), 7.25-7.28 (1H, m, ArH), 5.62 (2H, bs, NH_2), 2.44 (3H, s, CH_3). LCMS [ES+]: *m/z* 225 $[M+H]^+$ T_R = 3.0 min.

***N*-(Cyclohexylmethyl)-6-m-tolyl-[1,2,4]triazolo[1,5-a]pyridine-2-amine (36)**. 27 (50 mg, 0.22 mmol), $NaCNBH_3$ (1M soln in THF, 0.33 mmol) and cyclohexanecarbaldehyde (37 mg, 0.33 mmol) were heated in a microwave reactor for 10 min at 180 °C, the reaction quenched with H_2O, the THF removed in-vacuo and the residue portioned between EtOAc and H_20. The organic layer was dried over $MgSO_4$, filtered and the solvent removed *in-vacuo*. Purification with column chromatography eluting with ether afforded the desired product as a colourless oil (27 mg, 36 %). ^{1}H NMR (500 MHz, $CDCl_3$): 8.54 (1H, dd, ArH, *J* = 1.8 and 0.7 Hz), 7.63 (1H, dd, ArH, *J* = 9.1 and 1.9 Hz), 7.44 (1H, dd, ArH, *J* = 9.1 and 0.7 Hz), 7.37-7.40 (3H, m, ArH), 7.23 (1H, d, ArH, *J* = 6.8 Hz), 4.56 (1H, t, CH, *J* = 6.2 Hz), 3.30 (2H, t, CH_2, *J* = 6.5 Hz), 2.45 (3H, s, CH_3), 1.85-1.87 (2H, m, cyclohexyl-H), 1.73-1.78 (2H, m, cyclohexyl-H), 1.64-1.72 (2H, m, cyclohexyl-H), 1.16-1.19 (2H, m, cyclohexyl-H), 1.00-1.08 (2H, m, cyclohexyl-H). LCMS [ES+]: *m/z* 321 $[M+H]^+$ T_R = 3.9 min.

***N*-(6-m-Tolyl-[1,2,4]triazolo[1,5-a]pyridinyl)acetamide (37)**. Prepared according to general procedure B on a 0.45 mmol scale. Purification by column chromatography eluting with ether afforded the product as a white solid (40 mg, 33 %). ^{1}H NMR (500 MHz, DMSO): 10.83 (1H, s, NH), 9.21 (1H, s, ArH), 7.99 (1H, dd, ArH, *J* = 9.2 and 1.8 Hz), 7.75 (1H, d, ArH, *J* = 9.1 Hz), 7.64 (1H, s, ArH), 7.63-7.56 (1H, m, ArH), 7.41-7.38 (1H, m, ArH), 7.24 (1H, d, ArH, *J* = 7.4 Hz), 2.40 (3H, s, CH_3), 2.16 (3H, s, CH_3). LCMS [ES+]: *m/z* 267 $[M+H]^+$ T_R = 2.9-3.2 min.

***N*-(6-m-Tolyl-[1,2,4]triazolo[1,5-a]pyridinyl)tetrahydrofuran-2-carboxamide (38)**. Prepared according to general procedure B on a 0.45 mmol scale. Purification by column chromatography eluting with ether afforded the product as a clear oil (20 mg, 13 %). ^{1}H NMR (500 MHz, DMSO): 10.51 (1H, s, NH), 9.22 (1H, s, ArH), 8.01 (1H, dd, ArH, *J* = 9.2 and 1.8 Hz), 7.78 (1H, dd, ArH, *J* = 9.2 and 0.7 Hz), 7.64 (1H, s, ArH), 7.60 (1H, d, ArH, *J* = 8.1 Hz), 7.40 (1H, t, ArH, *J* = 7.6 Hz), 7.25 (1H, d, ArH, *J* = 7.5 Hz), 4.52 (1H, s, CH), 4.02-3.98 (1H, m, CH), 3.85-3.81 (1H, m, CH), 2.40 (3H, s, CH_3), 2.27-2.20 (1H, m, CH), 2.01-1.84 (3H, m, 3 x CH). LCMS [ES+]: *m/z* 323 $[M+H]^+$ T_R = 4.9-5.1 min.

***N*-(6-m-Tolyl-[1,2,4]triazolo[1,5-a]pyridinyl)tetrahydro-2H-pyran-4-carboxamide (39)**. Prepared according to general procedure B on a 0.45 mmol scale. Purification by column chromatography eluting with ether afforded the product as a white solid (91 mg, 60 %). ^{1}H NMR (500 MHz, DMSO): 10.99 (1H, s, NH), 9.18 (1H, s, ArH), 7.99 (1H, dd, ArH, *J* = 9.2 and 1.8 Hz), 7.75 (1H, d, ArH, *J* = 8.7 Hz), 7.63 (1H, s, ArH), 7.59 (1H, d, ArH, *J* = 8.3 Hz),

7.40 (1H, t, ArH, J = 7.6 Hz), 7.25 (1H, d, ArH, J = 7.5 Hz), 3.09-3.07 (1H, m, CH), 2.92-2.89 (1H, m, CH), 2.77-2.73 (1H, m, CH), 2.65-2.64 (1H, m, CH), 2.62-2.52 (1H, m, CH), 2.40 (3H, s, CH3), 1.92 (1H, m, NH), 1.67-1.59 (2H, m, CH), 1.47-1.45 (1H, m, CH), 1.24 (1H, s, CH). LCMS [ES+]: *m/z* 337 $[M+H]^+$ T_R = 3.1-3.4 min.

***N*-(6-m-Tolyl-[1,2,4]triazolo[1,5-a]pyridinyl)piperidine-3-carboxamide (40)**. Prepared according to general procedure B on a 0.44 mmol scale. Purification by column chromatography eluting with ether afforded the t-BOC protected product as a cream solid (18 mg, 10 %). Deprotection with TFA:DCM (0.5:1 mL) afforded the desired product (4 mg, 28 %). ^{1}H NMR (500 MHz, DMSO): 10.99 (1H, bs, NH), 9.18 (1H, s, ArH), 7.99 (1H, dd, ArH, J = 9.2 and 1.8 Hz), 7.75 (1H, d, ArH, J = 8.7 Hz), 7.63 (1H, s, ArH), 7.59 (1H, d, ArH, J = 8.7 Hz), 7.40 (1H, t, ArH, J = 7.6 Hz), 7.25 (1H, d, ArH, J = 7.6 Hz), 3.09-3.07 (1H, m, CH), 2.92-2.89 (1H, m, CH), 2.77-2.73 (1H, m, CH), 2.62-2.52 (1H, m, CH), 1.93-1.91 (1H, m, CH), 1.67-1.59 (2H, m, CH), 1.44 (1H, m, CH), 1.24 (1H, bs, CH). LCMS [ES+]: *m/z* 336 $[M+H]^+$ T_R = 3.2-3.4 min.

***N*-(6-m-Tolyl-[1,2,4]triazolo[1,5-a]pyridinyl)benzamide (41)**. Prepared according to general procedure A on a 0.15 mmol scale. Purification by column chromatography eluting with ether afforded the product as a colourless solid (25 mg, 51 %). 1H NMR (500 MHz, $CDCl_3$): 9.39 (1H, s, NH), 8.81 (1H, s, ArH), 8.03 (3H, d, ArH, J = 7.4 Hz), 7.77 (1H, dd, ArH, J = 9.2 and 1.8 Hz), 7.61(1H, t, ArH, J = 7.4 Hz), 7.53 (2H, t, ArH, J = 7.4 Hz), 7.48 (1H, d, ArH, J = 9.2 Hz), 7.37-7.42 (2H, m, ArH), 2.52 (3H, s, CH_3). LCMS [ES+]: *m/z* 329 $[M+H]^+$ T_R = 4.5-4.6 min.

***N*-(6-m-Tolyl-[1,2,4]triazolo[1,5-a]pyridinyl)cyclobutanecarboxamide (42)**. Prepared according to general procedure A on a 0.15 mmol scale from 27, purification by column chromatography eluting with ether afforded the product as a colourless solid (20 mg, 44 %). ^{1}H NMR (500 MHz, $CDCl_3$): 8.75 (1H, bs, NH), 8.11 (1H, bs, ArH), 7.76 (1H, dd, ArH, J = 9.2 and 1.8 Hz), 7.63 (1H, d, ArH, J = 9.2 Hz), 7.32-7.41 (1H, m, ArH), 7.22 (1H, d, ArH, J = 6.5 Hz), 2.46 (3H, s, CH_3), 2.05 (2H, m, cyclobutyl-H), 1.83 (2H, m, cyclobutyl-H), 1.30-1.45 (3H, m, cyclobutyl-H). LCMS [ES+]: *m/z* 307 $[M+H]^+$ T_R = 3.6 min.

***N*-(6-m-Tolyl-[1,2,4]triazolo[1,5-a]pyridinyl)cyclopentanecarboxamide (43)**. 3-Cyclopentanecarbonyl chloride (29 µL, 0.22 mmol) was added to a stirred solution of 27 (50 mg, 0.22 mmol), pyridine (anhydrous, 0.4 mL) and DMAP (2 mg, 0.02 mmol) at 0 °C. The reaction mixture was stirred at rt for 16 h diluted with DCM and washed with 15 % NaOH solution (2 x 10 mL), dried over $MgSO_4$, filtered and the solvent removed *in-vacuo*. Column chromatography eluting with ether afforded the desired product (14 mg, 20 %). ^{1}H NMR (500 MHz, $CDCl_3$): 8.55 (1H, s, ArH), 8.15 (1H, bs, ArH), 7.76 (1H, dd, ArH, J = 9.2 and 1.8 Hz), 7.62 (1H, d, ArH, J = 9.2 Hz), 7.36-7.40 (3H, m, ArH), 7.23-7.25 (1H, m, ArH), 2.84 (1H, bs, CH), 2.44 (3H, s, CH_3), 1.93-2.00 (4H, m, cyclopentyl-H), 1.77-1.85 (2H, m, cyclopentyl-H), 1.68-1.68 (2H, m, cyclopentyl-H). LCMS [ES+]: *m/z* 321 $[M+H]^+$ T_R = 3.5-3.6 min.

2-Cyclohexyl-*N*-(6-m-tolyl-[1,2,4]triazolo[1,5-a]pyridinyl)acetamide (45). Prepared following general procedure B on a 0.44 mmol scale to afford the desired product (24 mg, 16 %). ^{1}H NMR (500 MHz, $CDCl_3$): 10.76 (1H, bs, NH), 9.19 (1H, s, ArH), 7.98 (1H, d, ArH, J = 8.6 Hz), 7.74 (1H, d, ArH, J = 9.7 Hz), 7.63 (1H, s, ArH), 7.58 (1H, d, ArH, J = 8.2 Hz), 7.40 (1H, t, ArH, J = 7.0 Hz), 7.24 (1H, d, ArH, J = 8.2 Hz), 2.40 (3H, s, CH_3), 2.30 (1H, bs, CH), 1.63-1.72 (5H, cyclohexyl-H), 1.13-1.24 (4H, m, cyclohexyl), 0.94-0.99 (2H, m, cyclohexyl-H). LCMS [ES+]: *m/z* 349 $[M+H]^+$ T_R = 3.7-3.8 min.

3-Cyclopentyl-*N*-(6-m-tolyl-[1,2,4]triazolo[1,5-a]pyridinyl)propanamide (46). Prepared following general procedure B on a 0.44 mmol scale to afford the desired product (92 mg, 59 %). ^{1}H NMR (500 MHz, $CDCl_3$): 10.54 (1H, bs, NH), 8.95 (1H, s, ArH), 7.74 (1H, d, ArH, *J* = 9.0 Hz), 7.51 (1H, d, ArH, *J* = 9.0 Hz), 7.40 (1H, s, ArH), 7.35 (1H, d, ArH, *J* = 7.3 Hz), 7.16 (1H, t, ArH, *J* = 7.3 Hz), 7.00 (1H, d, ArH, *J* = 7.3 Hz), 2.16 (3H, s, CH_3), 1.52 (3H, bs, cyclohexyl-H), 1.37 (4H, bs, cyclohexyl-H), 1.25 (2H, bs, cyclohexyl-H), 0.87 (2H, bs, cyclohexyl-H). LCMS [ES+]: *m/z* 349 $[M+H]^+$ T_R = 3.5 min.

6-(3,5-Dimethylphenyl)-[1,2,4]triazolo[1,5-a]pyridin-3-amine (20b). 6-Bromo-[1,2,4]triazolo[1,5-a]pyridine-2-amine (0.63 g, 2.96 mmol) was suspended in DMF (5 mL) and K_2CO_3 (2M, 5 mL), $Pd(PPh_3)_4$ (0.1 g 0.09 mmol) and 3,5-dimethylboronic acid (0.75 g, 0.5 mmol) were heated at 160 °C for 5 min in µW reactor. The reaction was diluted with EtOAc washed with LiCl (5 % aq., 20 mL), NaOH (15 %, 20 mL) and H_2O (3 x 30 mL), dried over $MgSO_4$, filtered and the solvent removed *in-vacuo*. Column chromatography eluting with EtOAc afforded the desired product (136 mg, 24 %). 1H NMR (500 MHz, $CDCl_3$): 8.48 (1H, dd, ArH, *J* = 1.8 and 0.8 Hz), 7.64 (1H, dd, ArH, *J* = 9.1 and 1.8 Hz), 7.46 (1H, dd, ArH, *J* = 9.1 and 0.8 Hz), 7.16 (2H, s, ArH), 7.05 (1H, s, ArH), 4.47 (2H, bs, NH_2), 2.40 (6H, s, 2 x CH_3). LCMS [ES+]: *m/z* 239 $[M+H]^+$ T_R = 3.1-3.3 min.

***N*-(6-(3,5-Dimethylphenyl)-[1,2,4]triazolo[1,5-a]pyridinyl)-3-phenylpropanamide (47)**. Prepared following general procedure F on a 0.21 mmol scale. Column chromatography eluting with ether afforded the desired product (61 mg, 82 %). 1H NMR (500 MHz, $CDCl_3$): 8.72 (1H, bs, NH), 8.41 (1H, bs, ArH), 7.74 (1H, dd, ArH, *J* = 9.2 and 1.8 Hz), 7.61 (1H, d, ArH, *J* = 9.2 Hz), 7.27 – 7.30 (3H, m, ArH), 7.20-7.24 (2H, m, ArH), 7.18 (1H, s, ArH), 7.08 (1H, s, ArH), 3.12 (2H, t, CH_2, *J* = 7.7 Hz), 2.41 (6H, s, 2 x CH_3), 1.58 (2H, s, CH_2). LCMS [ES+]: *m/z* 371 $[M+H]^+$ T_R = 3.5-3.6 min.

***N*-(6-(3,5-Dimethylphenyl)-[1,2,4]triazolo[1,5-a]pyridinyl)cycloheptanecarboxamide (48)**. Prepared following general procedure F on a 0.21 mmol scale. Column chromatography eluting with ether afforded the desired product (36 mg, 50 %). 1H NMR (500 MHz, CDCl3): 9.16 (1H, bs, NH_2), 8.75 (1H, s, ArH), 7.77 (1H, dd, ArH, *J* = 9.1 and 1.8 Hz), 7.61 (1H, d, ArH, *J* = 9.1 Hz), 7.16 (2H, s, ArH), 7.07 (1H, s, ArH), 2.40 (6H, s, 2 x CH_3). 2.00-2.07 (2H, m, cycloheptyl-H), 1.84-1.97 (5H, m, cycloheptyl-H), 1.48-1.62 (7H, m, cycloheptyl-H). LCMS [ES+]: *m/z* 363 $[M+H]^+$ T_R = 2.9 min.

General procedure for the preparation of ureas 50, 52-61

1-(5,5-Dimethyl-3-oxocyclohex-1-en-1-yl)-3-phenylurea (50): 3-Amino-5,5-dimethylcyclohexeneone (100 mg, 0.72 mmol) was dissolved in 1,4-dioxane (3 ml) then phenylisocyanate (0.93 ml, 0.86 mmol) was added in one portion at room temperature. The reaction mixture was heated to 90 ºC with stirring for 16 h then cooled to room temperature. The resulting precipitate was collected, washed with 1,4-dioxane (5 ml) and diethyl ether (5 x 5 ml) and dried under vacuum to give **50** as a white solid (107 mg, 58 % yield). ^{1}H NMR (DMSO, 500 MHz) δ=8.91 (1H, s, NH), 8.66 (1H, s, NH), 7.44-7.41 (2H, m, ArH), 7.32-7.29 (2H, m, ArH), 7.04-7.01 (1H, m, ArH), 6.45 (1H, s, CH), 2.33 (2H, s, CH_2), 2.11 (2H, s, CH_2) and 1.02 (6H, s, Me). LCMS 97% (M+H, 259)

1-(Cyclohexylmethyl)-3-(5,5-dimethyl-3-oxocyclohex-1-en-1-yl)urea (53): Prepared following general procedure A to afford **53** as a white solid (61 mg, 31% yield). ^{1}H NMR (DMSO, 500 MHz) δ= 8.40 (1H, s, NH), 6.48 (1H, t, J=5.8*Hz*, NH), 6.39 (1H, s, CH), 2.90 (2H, t, J=6.3*Hz*, CH), 2.24 (2H, s, CH_2), 2.05 (2H, s, CH_2), 1.70-1.66 (5H, m, CH), 1.38-1.34 (1H, m, CH), 1.23-1.08 (3H, m, CH), 0.99 (6H, s, Me), and 0.91-0.83 (2H, m, CH). LCMS 98% (M+H, 279).

1-(5,5-Dimethyl-3-oxocyclohex-1-en-1-yl)-3-(2-methoxyphenyl)urea (55): Prepared following general procedure A to afford **55** as a white solid (114 mg, 55% yield). ^{1}H NMR (DMSO, 500 MHz) δ=9.24 (1H, s, NH), 8.51 (1H, s, NH), 8.04 (1H, dd, J=1.5 and 8.0*Hz*, ArH), 7.05-6.98 (2H, m, ArH), 6.93-6.89 (1H, m, ArH), 6.50 (1H, s, CH), 3.87 (3H, s, Me), 2.32 (2H, s, CH_2), 2.11 (2H, s, CH_2) and 1.03 (6H, s, Me). LCMS 100% (M+H, 289).

1-(5,5-Dimethyl-3-oxocyclohex-1-en-1-yl)-3-(4-methoxyphenyl)urea (57): Prepared following general procedure A to afford **57** as a white solid (113 mg, 50% yield). ^{1}H NMR (DMSO, 500 MHz) δ=8.71 (1H, s, NH), 8.58 (1H, s, NH), 7.33 (2H, d, J=8.7*Hz*, ArH), 6.89 (2H, d, J=8.7*Hz*, ArH), 6.43 (1H, s, CH), 3.72 (3H, s, Me), 2.32 (2H, s, CH_2), 2.10 (2H, s, CH_2) and 1.02 (6H, s, Me). LCMS 100% (M+H, 289).

1-(4-(Benzyloxy)phenyl)-3-(5,5-dimethyl-3-oxocyclohex-1-en-1-yl)urea (58): Prepared following general procedure A to afford **58** as a white solid (166 mg, 60% yield). ^{1}H NMR (DMSO, 500 MHz) δ=8.73 (1H, s, NH), 8.60 (1H, s, NH), 7.46-7.38 (7H, m, ArH), 7.35-7.31 (2H, m, ArH), 6.43 (1H, s, CH), 5.07 (2H, s, CH_2), 2.32 (2H, s, CH_2), 2.10 (2H, s, CH_2) and 1.02 (6H, s, Me). LCMS 96% (M+H, 365).

Methyl 4-(3-(5,5-dimethyl-3-oxocyclohex-1-en-1-yl)ureido)benzoate (59): Prepared following general procedure A to afford **59** as a white solid (101 mg, 44% yield). ^{1}H NMR (DMSO, 500 MHz) δ=9.31 (1H, s, NH), 8.77 (1H, s, NH), 7.91 (2H, d, J=8.8*Hz*, ArH), 7.57 (2H, d, J=8.8*Hz*, ArH), 6.45 (1H, s, CH), 3.82 (3H, s, Me), 2.35 (2H, s, CH_2), 2.12 (2H, s, CH_2) and 1.02 (6H, s, Me). LCMS 100% (M+H, 316).

1-(5,5-Dimethyl-3-oxocyclohex-1-en-1-yl)-3-(4-hydroxyphenyl)urea (60): Compound **12** (50 mg, 0.14 mmol) was dissolved in MeOH (8 ml) and water (2 ml) then $Pd(OH)_2$ (10 mg of 20% w/w on carbon) was added in one portion. The mixture was evacuated the back filled with Argon the process repeated then the flask evacuated and filled with hydrogen. The reaction was stirred vigorously for 20 hours then evacuated and back filled with nitrogen twice before opening the reaction to air. The reaction mixture was filtered through celite washing with MeOH (2 × 10 ml) then concentrated under vacuum. Triturating with hot Et_2O gave **60** as a white solid (11 mg, 0.40 mmol, 29% yield). ^{1}H NMR (DMSO, 500 MHz) δ=9.19 (1H, s), 8.61 (1H, s), 8.57 (1H, s), 7.20 (2H, d, J=8.7*Hz*, ArH), 6.70 (2H, d, J=8.7*Hz*, ArH), 6.42 (1H, s, CH), 2.31 (2H, s, CH_2), 2.09 (2H, s, CH_2) and 1.02 (6H, s, Me). LCMS 100% (M+H, 275).

1-(3-Oxocyclohex-1-en-1-yl)-3-phenylurea (61): Prepared following general procedure A to afford **61** as a white solid (147 mg, 71% yield). ^{1}H NMR (DMSO, 500 MHz) δ=8.98 (1H, s, NH), 8.74 (1H, s, NH), 7.43-7.41 (2H, m, ArH), 7.32-7.29 (2H, m, ArH), 7.04-7.01 (1H, m, ArH), 6.46 (1H, s, CH), 2.44 (2H, t, J=6.0*Hz*, CH_2), 2.23-2.20 (2H, m, CH_2) and 1.93-1.88 (2H, m, CH_2). LCMS 100% (M+H, 231).
